# A liver-infiltrating CD4^+^ Tfh1 cell response predicts HCV control, hepatitis, and seroconversion during acute infection

**DOI:** 10.1172/JCI178089

**Published:** 2025-09-16

**Authors:** Heather Blasczyk, William Bremer, Christopher C. Phelps, Yan Zhou, David G. Bowen, Zhaohui Xu, Robert Lanford, Naglaa H. Shoukry, Arash Grakoui, Nicole Skinner, Christopher M. Walker

**Affiliations:** 1 The Abigail Wexner Research Institute at Nationwide Children’s Hospital, Columbus, Ohio, USA.; 2 Texas Biomedical Research Foundation, San Antonio, Texas, USA.; 3 Centre de Recherche du Centre Hospitalier de l’Université de Montréal (CRCHUM) and Département de Médecine, Faculté de Médecine, Université de Montréal, Montréal, Québec, Canada.; 4 Emory Vaccine Center, Division of Microbiology and Immunology, Emory National Primate Research Center and Division of Infectious Diseases, Department of Medicine, Emory University School of Medicine, Atlanta, Georgia, USA.; 5 Department of Internal Medicine and; 6 Department of Pediatrics, College of Medicine, The Ohio State University, Columbus, Ohio, USA.

**Keywords:** Hepatology, Immunology, Cellular immune response, Hepatitis, T cells

## Abstract

Sustained CD4^+^ T cell immunity is required for resolution of acute hepatitis C virus (HCV) infection, but the response remains poorly characterized. Here, circulating CD4^+^ T cells with high programmed cell death 1 (PD-1) and ICOS coexpression were temporally associated with onset of virus control, seroconversion, and hepatitis in HCV-infected chimpanzees. Coproduction of T follicular helper (Tfh) (IL-21 and CXCL13) and Th1 (IFN-γ and TNF) cytokines after stimulation with HCV nonstructural proteins demonstrated that the response was predominately Tfh1 like and virus specific. Transcriptional analysis verified a Tfh1 lineage assignment. Effector-related genes such as *ADGRG1* (GPR56), *ZNF683* (Hobit), and *KLRB1* (CD161) were also expressed. HCV-specific PD-1^hi^ICOS^hi^ CD4^+^ Tfh1-like cells were enriched in liver, suggesting the potential for B and CD8^+^ T cell help at the site of virus replication. Most circulating and intrahepatic PD-1^hi^ICOS^hi^ CD4^+^ Tfh1-like cells did not express CXCR5 and therefore resembled CXCR5^–^CXCL13^+^ peripheral helper cells that infiltrate tumors and tissues inflamed by autoimmunity. PD-1^hi^ICOS^hi^ CD4^+^ Tfh1-like cells also peaked after hepatitis A virus infection, but the response was accelerated by several weeks compared with HCV infection. The PD-1^hi^ICOS^hi^ phenotype and temporal association between the peak response and alanine aminotransferase may provide markers to guide human studies of CD4^+^ T cell immunity against HCV and other hepatotropic viruses.

## Abstract 2

Immune responses the protect against persistent hepatitis C virus infection are poorly defined, complicating vaccine development. This study identifies a novel CD4+ T cell response at the site of virus replication.

## Graphical abstract

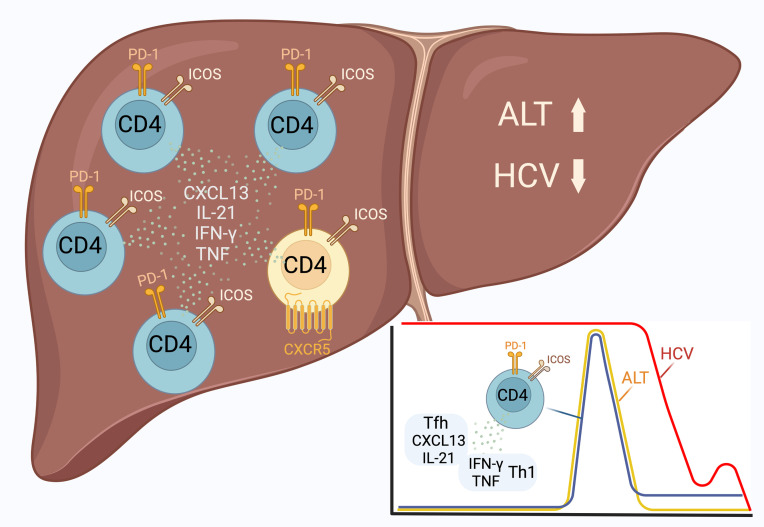

## Introduction

Only 25%–30% of human hepatitis C virus (HCV) infections resolve spontaneously (1, 2). This outcome requires robust neutralizing antibody and CD8^+^ T cell responses that are highly dependent on sustained help, possibly from 3 distinct CD4^+^ T cell subsets (3–5). CD4^+^ Th1 cells that produce IFN-γ and TNF are a well-established feature of HCV infections that resolve (6–9). CD4^+^ Th17-like cells have also been associated with robust CD8^+^ T cell immunity and resolution of infection (10). More recently, CD4^+^ T follicular helper 1 (Tfh1) cells defined by expression of Tfh (programmed cell death 1 [PD-1], ICOS, and CXCR5) and Th1 (CXCR3) markers were detected in blood during acute primary HCV infection (11–13), after reinfection with the virus (14), and during direct-acting antiviral (DAA) treatment of chronic infection (15). Onset of the Tfh1-like response during acute infection coincided with expansion of HCV-specific B cells and seroconversion (11–13). The relationship between the Th1, Th17, and Tfh1-like CD4^+^ T cell subsets observed during acute HCV infection is not well defined. Partial overlap in function, for instance, production of IFN-γ and IL-21 by the Th17 and Tfh1-like subsets (10–13), suggests the potential for a close lineage relationship or plasticity during CD4^+^ T cell differentiation. Transcriptional and phenotypic analysis of CD4^+^ T cells during acute infection could help define the scope of protective helper responses in humans, but this remains challenging. CD4^+^ T cells target multiple HCV epitopes and circulate at low frequency, hampering class II tetramer visualization and enrichment. Surrogate markers for direct ex vivo identification of HCV-specific CD4^+^ T cells have not been established. Sampling of blood and liver for analysis of CD4^+^ T cell immunity is also complicated because acute hepatitis C is often clinically silent and the onset of adaptive immunity occurs over a wide and unpredictable time frame of several weeks to months after infection.

Studies of HCV-infected chimpanzees also provided insight into protective T cell immunity (16). As in humans, the CD4^+^ T cell response is broad, multifunctional, and susceptible to premature failure that results in chronic infection (16). Moreover, antibody-mediated depletion of CD4^+^ T cells from chimpanzees with naturally acquired protective immunity caused CD8^+^ T cell failure and persistence after HCV reinfection (17). Although this model is no longer available for research, cryopreserved PBMCs and liver mononuclear cells (LMCs) from completed studies provided an opportunity to characterize CD4^+^ T cell immunity during acute HCV infection. CD4^+^ T cells defined by high coexpression of PD-1 and ICOS expanded in blood. The response peaked with acute hepatitis and seroconversion, immediately before a sharp initial decline in viremia. PD-1^hi^ICOS^hi^ CD4^+^ [CD4^P/I(hi)^] T cells were predominately HCV specific and had a Tfh1-like transcriptional and functional profile. Most CD4^P/I(hi)^ T cells did not express CXCR5, the chemokine receptor required for Tfh cell homing to secondary lymphoid organs. The cells were highly enriched in the liver, suggesting the potential to direct formation of hepatic lymphoid structures that are an unexplained feature of HCV infection and to regulate B and CD8^+^ T cells at the site of virus replication.

## Results

### CD4^P/I(hi)^ T cell expansion and control of HCV replication.

**Figure 1:**
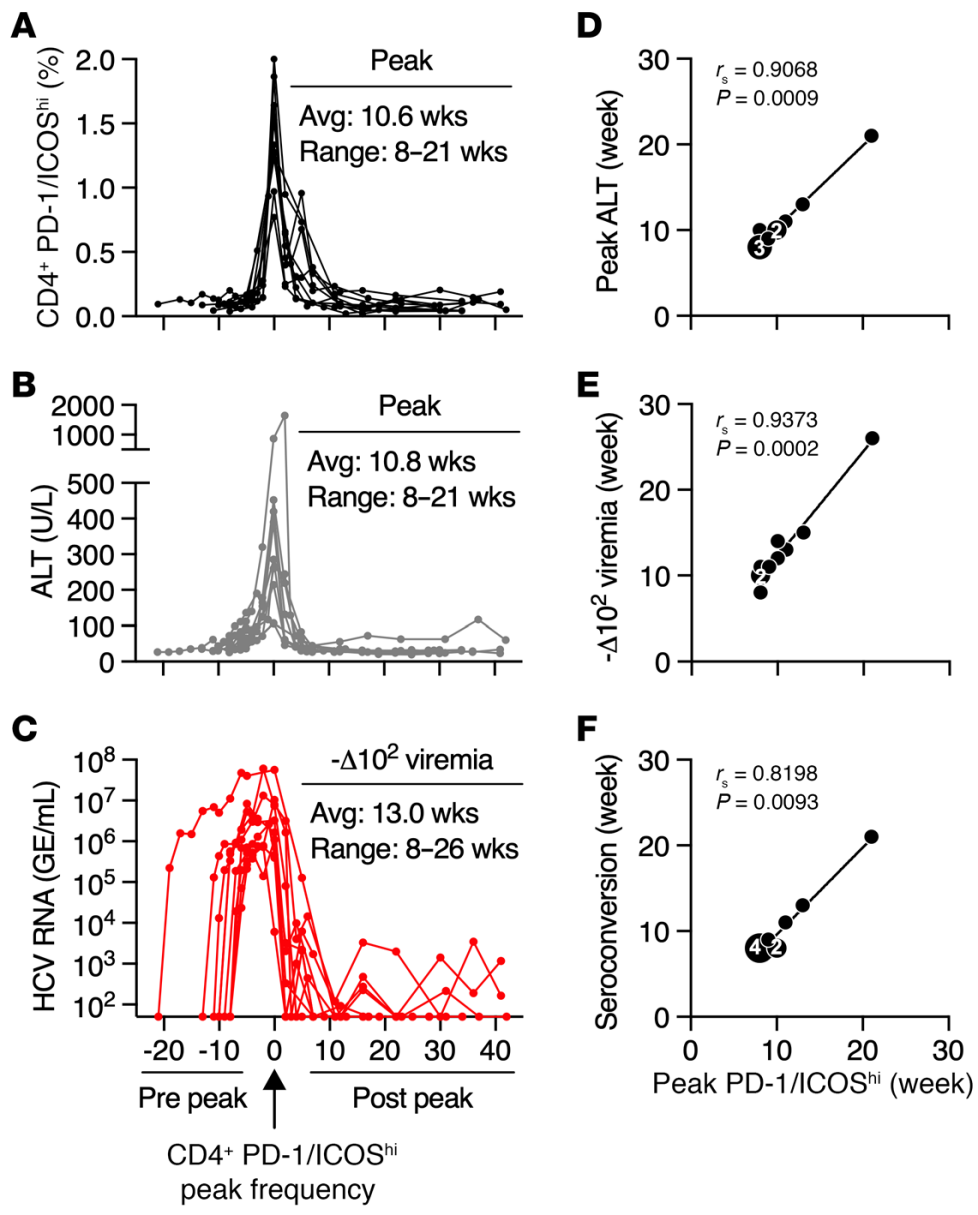
Acute HCV infection profile. Temporal alignment of circulating (**A**) CD4^P/I(hi)^ T cells, (**B**) ALT, and (**C**) HCV serum RNA titer by peak CD4^P/I(hi)^ T cell frequency in 10 HCV genotype 1a–infected animals. The CD4^P/I(hi)^ T cell gate is shown for a representative animal (Supplemental Figure 2A). The week when CD4^P/I(hi)^ T cell frequencies peaked (*x* axis) is plotted against the week of (**D**) peak ALT, (**E**) >2 log_10_ decline in HCV viremia, and (**F**) HCV core and NS protein seroconversion (*y* axis). Numbers within symbols indicate overlapping data points. Significance was determined using Spearman’s rank correlation with *P* values shown.

CD4^+^ T cell immunity was assessed in 10 chimpanzees during acute HCV genotype 1a infection. Eight animals cleared the infection over 10–12 months of follow-up (Supplemental Figure 1, A–H; supplemental material available online with this article; https://doi.org/10.1172/JCI178089DS1). Two had low fluctuating viremia that persisted through termination of the study (Supplemental Figure 1, I and J). CD4^+^ T cells with high coexpression of the PD-1 and ICOS activation markers increased in frequency from approximately 0.1% at baseline to a peak of approximately 1%–2% between weeks 8 and 21 after infection (Supplemental Figure 1, A–J). Alignment of circulating CD4^P/I(hi)^ T cells from 10 animals by peak frequency (Figure 1A) revealed a temporal kinetic association with peak alanine aminotransferase (ALT) (Figure 1B) and initial control of viremia (Figure 1C). CD4^P/I(hi)^ T cells and serum ALT titers peaked at approximately the same time (average of 10.6 and 10.8 weeks after infection, respectively) (Figure 1, A and B) and were significantly correlated (Spearman’s *r* [*r*_s_] = 0.907, *P* ≤ 0.001) (Figure 1D). Initial control of virus replication, defined as a 2 log_10_ decline in serum HCV RNA titer, was observed approximately 2–3 weeks after the CD4^P/I(hi)^ T cell peak (average 13 weeks, range 8–26 weeks; *r*_s_ = 0.937, *P* ≤ 0.001) (Figure 1, C and E). This CD4^+^ T cell response also peaked in close temporal proximity to seroconversion against the HCV core and nonstructural (NS) proteins (average 10.6 versus 10.9 weeks, respectively; *r*_s_ = 0.820, *P* ≤ 0.01) (Figure 1F and Supplemental Figure 1K).

**Figure 2:**
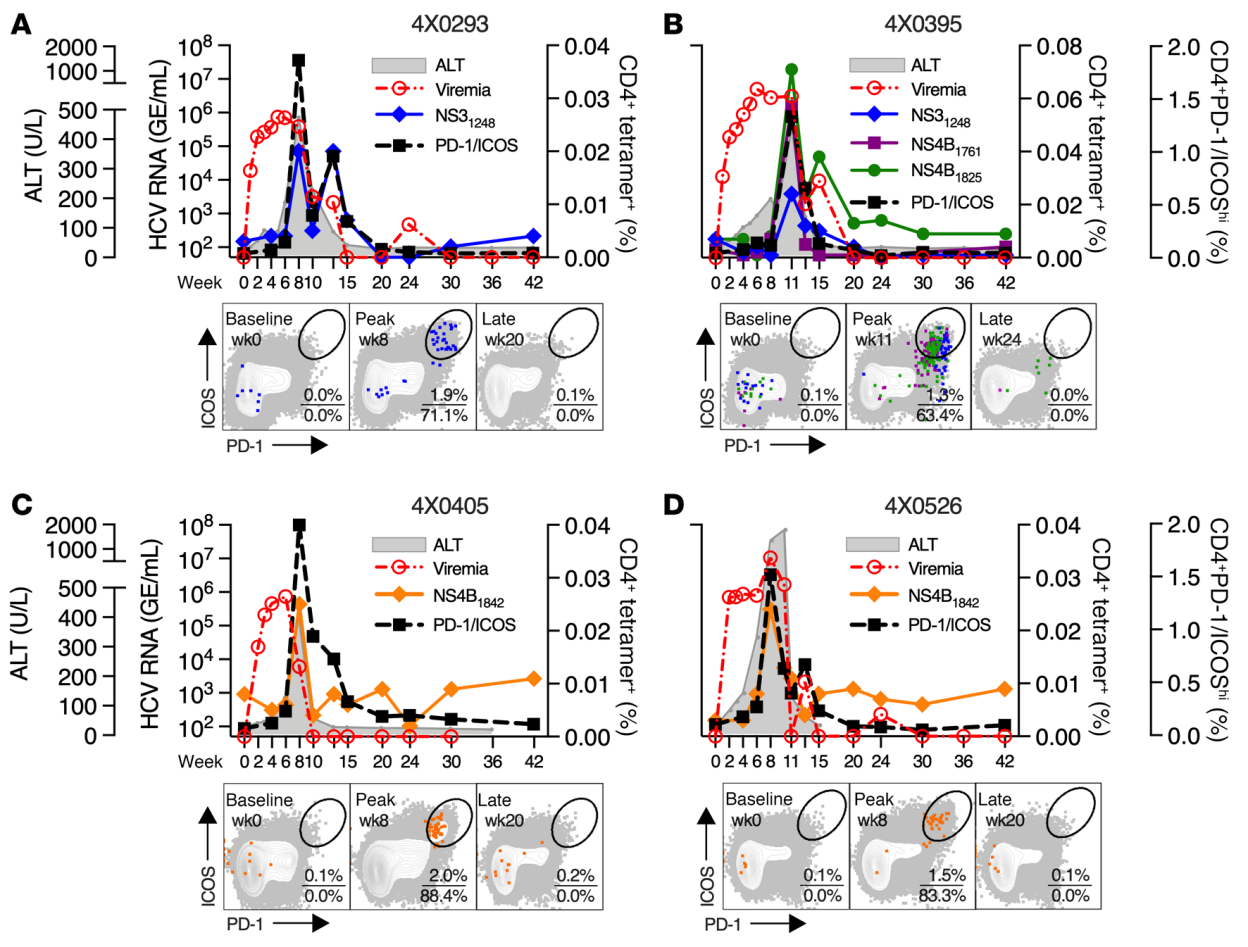
HCV class II tetramer–positive CD4^+^ T cells. (**A**–**D**) Circulating CD4^+^ T cells with a PD-1^hi^ICOS^hi^ phenotype or positive staining with HCV class II tetramers measured in 4 animals at the indicated time points. HCV serum RNA and ALT titers are also shown for reference. Class II tetramer–positive CD4^+^ T cell gating is shown in Supplemental Figure 2A. HCV class II tetramer–positive CD4^+^ T cells (colored dots) are overlaid on circulating CD4^+^ T cells also stained for PD-1 and ICOS expression at the indicated week and at all time points through week 42 for a representative animal (4X0395) in Supplemental Figure 2B. The percentage of circulating (top number) and class II tetramer–positive (bottom number) CD4^+^ T cells within the PD-1^hi^ICOS^hi^ gate is provided.

Temporal overlap of CD4^P/I(hi)^ T cell expansion, hepatitis, and seroconversion suggested that the response was HCV specific. This possibility was supported by a close kinetic relationship between CD4^+^ T cell responses measured by the PD-1^hi^ICOS^hi^ phenotype and by staining with *Pan troglodytes* (*Patr*) class II tetramers (Supplemental Table 1) containing HCV NS3 or NS4 epitopes (Figure 2). Responses measured by both approaches peaked at week 8 (4X0293, 4X0405, and 4X0526) or week 11 (4X0395) after infection and contracted in parallel with a sharp drop in viremia (Figure 2). Moreover, most class II tetramer–positive cells (~60%–90%) were located within the PD-1^hi^ICOS^hi^ gate at these peak time points (Figure 2). These observations suggested that the peak CD4^P/I(hi)^ T cell response was perhaps largely HCV specific. However, peak CD4^P/I(hi)^ T cell frequencies (~1.3%–2.0%) were considerably higher than those measured with the small panel of available class II tetramers (~0.02%–0.08%) (Supplemental Figure 2C), as predicted for a broad acute-phase response that can, in some individuals, target more than 20 discrete class II epitopes (6).

### Transcriptional signature of CD4^P/I(hi)^ T cells.

High PD-1 and ICOS coexpression could provide a surrogate marker for transcriptional and phenotypic analysis of HCV-specific CD4^+^ T cells without the limitations imposed by class II tetramers. RNA-Seq was undertaken to further define CD4^P/I(hi)^ T cells that expanded in response to HCV infection. Circulating CD4^P/I(hi)^, CD4^P/I(int)^, and CD4^P/I(lo)^ T cells were sorted from 5 animals (4X0526, 4X0405, 4X0339, 4X0312, and 4X0395) at the peak of the CD4^P/I(hi)^ response (Supplemental Figure 1, B–F) using gates shown in Supplemental Figure 3A. All 3 populations yielded sufficient RNA for sequencing except for the CD4^P/I(lo)^ and CD4^P/I(int)^ T cells from animals 4X0312 and 4X0339, respectively, and were transcriptionally distinct by principal component analysis (Supplemental Figure 3B). Differentially expressed genes (DEGs) were identified by pairwise comparison of the effector CD4^P/I(hi)^ and CD4^P/I(int)^ populations with CD4^P/I(lo)^ T cells that were provisionally defined as naive (e.g., CCR7^+^CD45RA^+^) by immunostaining (Supplemental Figure 3, C and D). The CD4^P/I(hi)^ versus CD4^P/I(lo)^ T cell comparison yielded 1,712 DEGs (702 downregulated and 1,010 upregulated) (Supplemental Figure 3E). The CD4^P/I(int)^ T cells versus CD4^P/I(lo)^ comparison yielded 828 DEGs (249 downregulated and 579 upregulated) (Supplemental Figure 3E). The top 50 upregulated (Supplemental Table 2A) and downregulated (Supplemental Table 2B) DEGs in each comparison were identified by ranking for log_2_ fold change followed by adjusted *P* value (padj).

**Figure 3:**
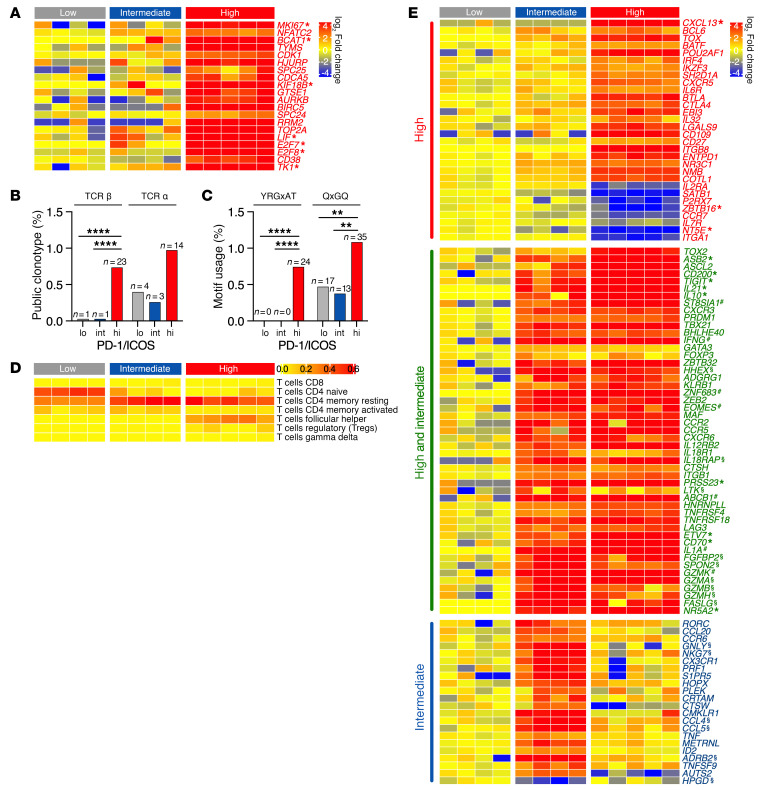
CD4^+^ T cell transcriptional signatures. (**A**) DEGs associated with T cell proliferation and activation. Those among the top 50 upregulated by CD4^P/I(hi)^ T cells are indicated (*). (**B**) TcR clonotypes (%) with public TCRβ and TCRα chains for CD4^+^ T cells with low, intermediate, and high PD-1 and ICOS expression. (**C**) Frequency of dominant public CDR3 motifs (YRGxAT and QxGQ, single aa code). Significance (**B** and **C**) was determined by Fisher’s exact test with Benjamini-Hochberg correction applied for multiple comparisons. **P < 0.01, ****P < 0.0001. (**D**) Heatmap of LM22 CIBERSORT T cell gene modules in CD4^+^ T cells sorted for low, intermediate, and high PD-1 and ICOS expression. (**E**) Immune-related genes significantly upregulated (log_2_ fold change >1.5; padj < 0.05) by CD4^P/I(hi)^, CD4^P/I(hi)^ and CD4^P/I(int)^, or CD4^P/I(int)^ populations when compared with CD4^P/I(lo)^ T cells. *GATA3* and *FOXP3* that define Th2 and Treg subsets were not significant in any comparison. The top 50 up- or downregulated genes for CD4^P/I(hi)^ T cells (*), CD4^P/I(int)^ T cells (§), or both populations (#), when ranked sequentially by log_2_ fold change and then padj, are indicated.

The CD4^P/I(hi)^ T cell transcriptional signature was consistent with a response to infection. They were distinguished from sorted CD4^P/I(int)^ and CD4^P/I(lo)^ T cells by the proliferation, activation, and signaling modules of the blood transcriptome matrix (Supplemental Figure 3F) (18) and by upregulation of multiple genes associated with an activated state, such as *MKI67*, as well as *NFATC2* and *BCAT1*, which respond to upstream T cell receptor (TcR) signaling (Figure 3A) (19–22). CD4^P/I(hi)^ T cells were also enriched in genes encoding public TcR clonotypes, as expected for a response to viral antigens (23). TcR vβ public clonotypes, defined as clones with the same *TRBV* and *TRBJ* genes and identical β chain complementarity determining region 3 (CDR3) sequences, were significantly increased in CD4^P/I(hi)^ T cells when compared with CD4^P/I(int)^ and CD4^P/I(lo)^ T cells (Figure 3B). Visual comparison of CDR3 sequences in the TcR β chain public clonotypes identified 2 potential amino acid motifs, YRGxAT and QxGQ, that were defined prior to statistical testing and were the only 2 motifs tested. Alignment of TcR β chain CDR3 sequences (for example, Supplemental Figure 3G) confirmed significant usage of both motifs among CD4^P/I(hi)^ T cell clonotypes. For instance, motif YRGxAT was found in 24 CD4^P/I(hi)^ T cell clonotypes and 0 CD4^P/I(lo)^ and CD4^P/I(int)^ clonotypes (Figure 3C).

A transcriptional signature for CD4^P/I(hi)^ T cells was further defined by analysis of immune-related DEGs. CIBERSORT, a computational tool with gene modules for deconvolution of 7 activated and resting T cell populations (24), verified that CD4^P/I(lo)^ T cells were naive (Figure 3D). This assignment was further supported by differential expression of naive genes such as *SELL*, *SCML1*, *FHIT*, *PECAM1*, and *LEF1* (Supplemental Figure 3H) (19, 21, 25, 26). CD4^P/I(int)^ T cells were, along with the CD4^P/I(lo)^ and CD4^P/I(hi)^ populations, enriched in genes that comprise a resting memory module but were not otherwise defined by CIBERSORT (Figure 3D). CD4^P/I(hi)^ T cells that expanded in response to HCV infection were uniquely identified by the CIBERSORT follicular helper module (Figure 3D). A Tfh designation for CD4^P/I(hi)^ T cells was further supported by upregulation of *CXCL13*; transcription factors *BCL6*, *TOX*, *BATF*, *POU2AF1*, *IRF4*, and *IKZF3*; the SLAM-associated gene *SH2D1A*; chemokine receptor *CXCR5*; and *IL6R* required for IL-6–driven ICOS expression (27, 28) (Figure 3E). Coinhibitory receptor genes *BTLA* and *CTLA4* were upregulated (Figure 3E), but others associated with exhaustion, including *HAVCR2* (T cell immunoglobulin mucin receptor 3 [TIM-3]), *KLRG1*, *CD244* (2B4), and *CD160*, were not (log_2_ fold change < 1.5 and padj > 0.05), as described for CD4^+^ T cells in humans with acute resolving HCV infection (29, 30).

CD4^P/I(hi)^ T cells also expressed several genes not typical of the Tfh subset, including *EBI3*, a subunit of the IL-27, IL-35, and IL-39 cytokines (31), and the inflammatory cytokine *IL32* associated with liver injury in chronic hepatitis C (Figure 3E) (32). Upregulation of *LGALS9* (Figure 3E) was notable because the encoded galectin 9 (Gal-9) protein contributes to exhaustion of CD8^+^ T cells that express its TIM-3 (*HAVCR2*) receptor (10, 33, 34). Serum Gal-9 and CXCL13 titers increased as CD4^P/I(hi)^ T cells expanded in blood (Supplemental Figure 4, A–D). Both were significantly associated with the frequency of circulating CD4^P/I(hi)^ T cells (Supplemental Figure 4, E and F). CD4^P/I(hi)^ T cells also expressed *CD109*, which marks activated dengue virus–specific CD4^+^ T cells (35); costimulatory receptor *CD27*; integrin *ITGB8*; and the ectonucleotidase *ENTPD1* (CD39) (Figure 3E). Other upregulated genes included *NR3C1*, *NMB*, and *COTL1* (Figure 3E), which are coordinately expressed with *PDCD1*, *CXCL13*, and *ENTPD1* by tumor infiltrating CD4^+^ T cells (36, 37). CD4^P/I(hi)^ T cells downregulated genes that inhibit Tfh differentiation (*IL2RA* and *SATB1*) or pathogenic function (*P2RX7*) and promote Th17 development (*ZBTB16*) (Figure 3E) (38–43).

The CD4^P/I(hi)^ T cell transcriptional profile also comprised immune-related genes that had a similar pattern of differential expression in CD4^P/I(int)^ T cells (Figure 3E). Four genes associated with the Tfh lineage (*CD200*, *TIGIT*, *IL21*, and *IL10*) were upregulated by both sorted populations (Figure 3E) but were top 50 DEGs for CD4^P/I(hi)^ T cells only (Supplemental Table 2A), consistent with their dominant Tfh signature. Both populations also had a Th1 transcriptional profile defined by an equivalent fold increase in *CXCR3*; the *PRDM1*, *TBX21*, and *BHLHE40* transcription factors; and *IFNG*, a Th1 cytokine that was a top 50 DEG for CD4^P/I(hi)^ and CD4^P/I(int)^ T cells (Figure 3E and Supplemental Table 2A). CD4^P/I(hi)^ T cell assignment to these lineages was confirmed by immunostaining (Supplemental Figure 5). Master regulators of Tfh (Bcl-6) and non-Tfh (Blimp-1 encoded by *PRDM1*) differentiation, and associated Th1 (T-bet encoded by *TBX21*) and Tfh or Th17 (c-Maf encoded by *MAF*) transcription factors, were significantly increased in CD4^P/I(hi)^ versus CD4^P/I(lo)^ T cells (Supplemental Figure 5, C–F). CD4^P/I(hi)^ and CD4^P/I(int)^ T cells were also assessed for differential expression of genes that define other Th subsets. CD4^P/I(int)^ T cells had a Th17/Th1 (Th17/1) transcriptional bias because they also upregulated the Th17-related genes *RORC*, *CCL20*, and *CCR6* (Figure 3E). Th2 (*GATA3*) and Treg (*FOXP3*) transcription factor genes were not differentially expressed by CD4^P/I(hi)^ or CD4^P/I(int)^ T cells, consistent with upregulation of their *ZBTB32* and *HHEX* repressors (Figure 3E) (44, 45) and an absence of Th2 (*IL4*, *IL5*, and *IL13*) and Treg (*TGFB*) cytokine gene activity (log_2_ fold change < 1.5 and/or padj > 0.05).

Upregulation of other immune-related genes by CD4^P/I(hi)^ and CD4^P/I(int)^ T cells indicated overlap in effector/memory differentiation status, consistent with common central memory (CCR7^+^CD45RA^–^) and/or effector memory (CCR7^–^CD45RA^–^) phenotypes determined by immunostaining (Supplemental Figure 3, B and C). Both populations expressed *ADGRG1*, which encodes GPR56, a marker of effector memory CD4^+^ T cells in virus infections (46–48) and autoimmune diseases (20, 49). CD4^P/I(hi)^ and CD4^P/I(int)^ T cells also upregulated *KLRB1* (CD161), which delineates a population of proinflammatory CD4^+^ T cells that infiltrate the liver during chronic HCV infection (Figure 3E) (50). Multiple additional genes associated with *ADGRG1* and/or *KLRB1* expression were upregulated, including transcription factors (*ZNF683*, *ZEB2*, *EOMES*, and *MAF*); chemokine (*CCR2*, *CCR5*, and *CXCR6*) and cytokine (*IL12RB2*, *IL18R1*, and *IL18RAP*) receptors; cathepsin H (*CTSH*); integrin (*ITGB1*); serine protease (*PRSS23*); tyrosine kinase (*LTK*); the ATP-dependent efflux pump *ABCB1* (MDR1); *HNRNPLL*, which regulates CD45 splicing; and receptors *TNFRSF4* (OX40), *TNFRSF18* (GITR), and *LAG3* (Figure 3E) (46, 47, 51). The inflammatory *IL1A* cytokine gene that is expressed by effector/memory populations (52) was a top 50 DEG for CD4^P/I(hi)^ and CD4^P/I(int)^ T cells (Figure 3E and Supplemental Table 2A).

Several genes that are markers (*FGFBP2* and *SPON2*) or mediators (*GZMA*, *GZMB*, *GZMH*, and *FASLG*) of cytotoxic CD4^+^ T cell function were upregulated by CD4^P/I(hi)^ and CD4^P/I(int)^ T cells (Figure 3E). CD4^P/I(int)^ T cells had a more pronounced cytotoxic signature because these DEGs were, collectively, amongst the top 50 upregulated by this sorted population only (Supplemental Table 2A). Moreover, CD4^P/I(int)^ T cells, but not CD4^P/I(hi)^ T cells, were enriched in other cytotoxic/effector memory transcripts identified in human CMV (47, 53–55) and dengue virus–specific (46, 56) CD4^+^ T cells, including *GNLY*, *NKG7*, *CX3CR1*, *PRF1*, *S1PR5*, *HOPX*, *PLEK*, *CRTAM*, *CTSW*, *CMKLR1*, *CCL4*, *CCL5*, and the cytokines *TNF* and *METRNL* (IL-41) (Figure 3E and Supplemental Table 2A for *GNLY*, *NKG7*, *CCL4*, and *CCL5*). A unique transcriptional signature for Th17/1-like CD4^P/I(int)^ T cells was further defined by upregulation of transcriptional repressor *ID2*, the β-2 adrenergic receptor *ADRB2*, the *TNFSF9* 4-1BB ligand, and *AUTS2*, a transcription activator associated with the Th17 subset and *KLRB1* (CD161) expression (26, 51) (Figure 3E). *HPGD*, associated with the Treg subset (57), was downregulated when compared with CD4^P/I(lo)^ and CD4^P/I(hi)^ T cells (Figure 3E).

In summary, CD4^P/I(hi)^ and CD4^P/I(int)^ T cells had Tfh1 and cytotoxic Th17/1 transcriptional signatures, respectively. Both populations upregulated genes encoding IFN-γ and IL-21. CD4^P/I(hi)^ T cells that expanded only in response to HCV infection were enriched in public TcR clonotypes. They were also unique in upregulation of *CXCL13* and genes associated with activation, proliferation, and signaling typically initiated by TcR stimulation.

### CD4^P/I(hi)^ T cell function and antigen specificity.

**Figure 4:**
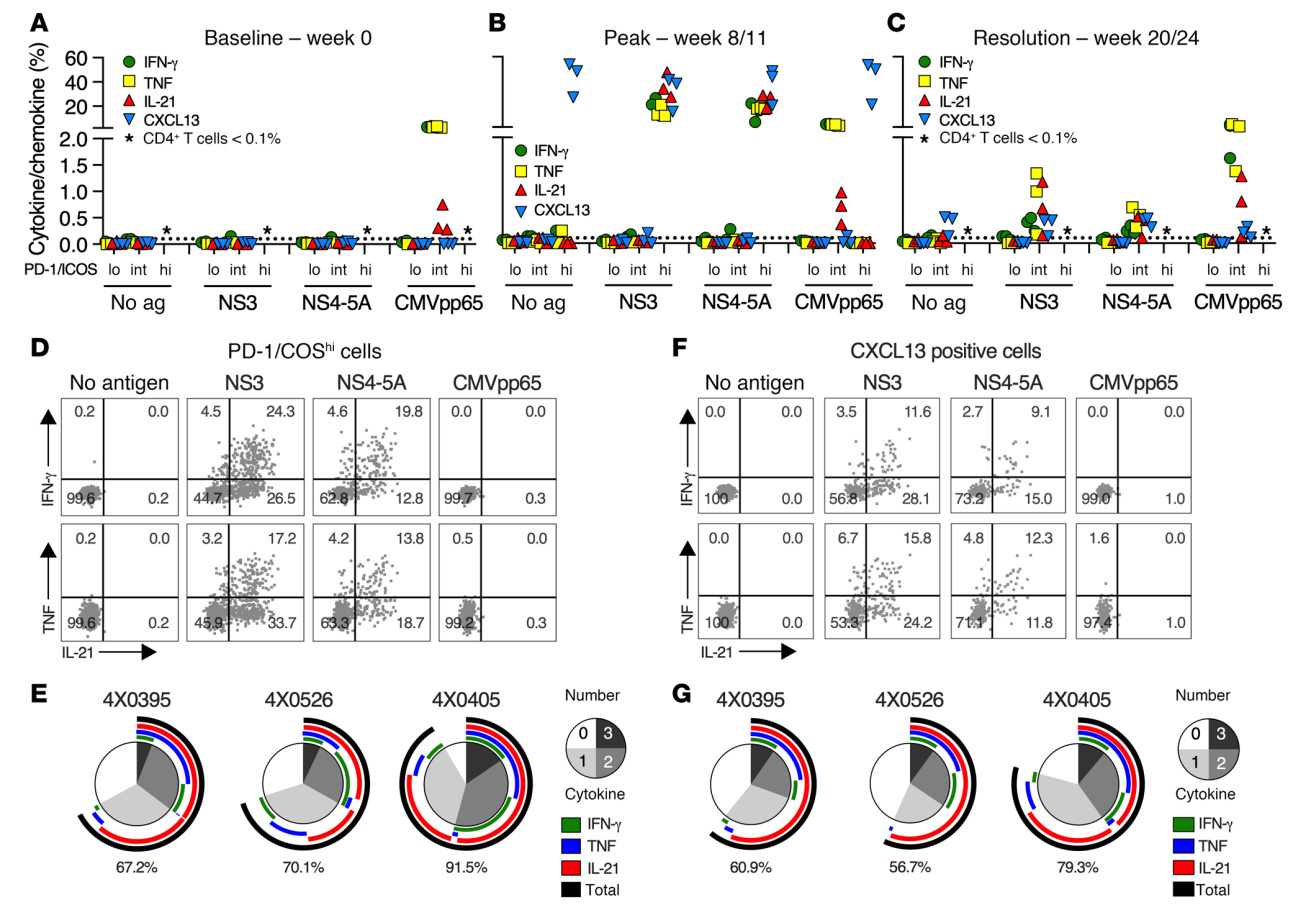
Function and HCV specificity of circulating CD4^+^ T cells. PBMCs from 3 animals (4X0395, 4X0405, and 4X0526) were stimulated with indicated peptide pools at (**A**) baseline, (**B**) the CD4^P/I(hi)^ T cell peak, and (**C**) after apparent resolution of viremia. Dot plots from a representative animal are shown in Supplemental Figure 6, B–D, for all 3 time points. The percentage of cytokine- and chemokine-producing CD4^+^ T cells with low, intermediate, and high PD-1 and ICOS expression is shown. Symbols represent individual animals. Asterisks indicate less than 0.1% of circulating CD4^+^ T cells (<25 cells) within the indicated PD-1/ICOS gate, a frequency too low for ICS analysis. (**D**) Dot plots show antigen-stimulated cytokine production by peak gated CD4^P/I(hi)^ T cells for the representative animal 4X0405. (**E**) The number (pie) and combination (arc) of cytokines produced by CD4^P/I(hi)^ T cells, summed for NS3 and NS4-5A antigen stimulation at the peak of response. Percentage of HCV antigen–stimulated CD4^P/I(hi)^ T cells producing at least 1 of the indicated cytokines is represented by the black arc and numerical value. (**F**) Cytokine coproduction by gated CXCL13^+^CD4^+^ T cells at the peak CD4 response from 4X0405 after stimulation with the indicated antigens. (**G**) The number (pie) and combination (arc) of cytokines coproduced by CXCL13^+^CD4^+^ T cells summed for NS3 and NS4-5A HCV antigen stimulation. Black arc and numerical value indicate the percentage of CXCL13^+^CD4^+^ T cells that coproduced at least 1 cytokine.

Tfh1 function and HCV specificity of circulating CD4^P/I(hi)^ T cells was assessed by intracellular cytokine staining (ICS) before infection (Figure 4A) at week 8 (4X0405 and 4X0526) or 11 (4X0395) when the response peaked (Figure 4B) and at week 20 or 24 after apparent resolution of infection (Figure 4C). ICS was undertaken after stimulation of PBMCs with HCV NS3 and NS4-NS5A or the pp65 antigen of chimpanzee CMV (chCMV) that naturally infects most animals (Figure 4, A–C). NS3 and NS4-NS5A were confirmed as dominant targets of the CD4^+^ T cell response (Supplemental Figure 6A). An HCV-specific CD4^+^ T cell response was not detected before infection (week 0) (Figure 4A). HCV antigen–stimulated CD4^+^ T cells produced IL-21, IFN-γ, and TNF at the week 8 or 11 peak (Figure 4B) and at lower frequencies at week 20 or 24 after infection (Figure 4C). chCMV pp65–specific CD4^+^ T cells were detected by ICS at all 3 time points (Figure 4, A–C), as expected for a memory response.

The cytokine response elicited by NS3 and NS4-5A was restricted to CD4^P/I(hi)^ T cells at the peak of the response (Figure 4B). Individual cytokines (IL-21, TNF, or IFN-γ) were detected in a high frequency (range of ~5%–40%) of CD4^P/I(hi)^ T cells when stimulated with either antigen, compared with less than 0.1% of CD4^P/I(lo)^ and CD4^P/I(int)^ T cells (Figure 4B). IL-21 was the dominant cytokine, produced most often in combination with IFN-γ and/or TNF, as expected for a Tfh1 response (Figure 4, D and E). Few CD4^+^ T cells produced Th1 cytokines (IFN-γ and TNF) without IL-21. This analysis also demonstrated that a majority of CD4^P/I(hi)^ T cells were HCV specific. Approximately 70%–90% of CD4^P/I(hi)^ T cells from animals 4X0395, 4X0526, and 4X0405 produced at least 1 cytokine in response to NS3 and NS4-5A (Figure 4E).

CD4^P/I(hi)^ T cells also produced CXCL13 after (Figure 4B) but not before (Figure 4A) HCV infection. CXCL13^+^ CD4^+^ T cells were detected under all stimulation conditions, including those with no antigen or pp65 control antigen and therefore differed from IL-21, IFN-γ, and TNF responses that were strictly HCV antigen dependent (Figure 4B). Most CXCL13^+^ CD4^P/I(hi)^ T cells were nonetheless HCV specific because the majority (60.9%, 4X0395; 56.7%, 4X0526; 79.3%, 4X0405) coproduced at least 1 other cytokine (IL-21, IFN-γ, and/or TNF) after NS3-NS5A but not chCMV pp65 antigen stimulation (Figure 4, F and G). IL-21 was the dominant cytokine coproduced by CXCL13^+^ CD4^+^ T cells, and a subset expressed all 4 Tfh (IL-21 and CXCL13) and Th1 (IFN-γ and TNF) cytokines (Figure 4G).

HCV-specific CD4^P/I(hi)^ T cells were distinct from chCMV-specific CD4^+^ T cells at the peak of the response. chCMV-specific T cells retained a PD-1^int^ICOS^int^ phenotype at all 3 time points and did not produce CXCL13, either spontaneously or after pp65 stimulation (Figure 4, A–C). Moreover, the frequency of chCMV-specific CD4^+^ T cells that produced IL-21 after pp65 stimulation was comparatively low when compared with Th1 cytokines IFN-γ and TNF (Figure 4, A–C). These observations indicate that bystander chCMV pp65–specific CD4^+^ T cells did not acquire a full Tfh1 functional profile or PD-1^hi^ICOS^hi^ phenotype that defined HCV-specific CD4^P/I(hi)^ T cells at the peak of the response to infection. Functional HCV-specific CD4^+^ T cells remained in circulation at week 20 or 24 but at low frequency compared with the peak (Figure 4, B and C). Production of IL-21, IFN-γ, and TNF, but not CXCL13, required NS3 and NS4-5A stimulation (Figure 4C), as observed at the peak of the response (Figure 4B). Importantly, cytokine-positive CD4^+^ T cells shifted from a PD-1^hi^ICOS^hi^ phenotype at the peak (Figure 4B) to a PD-1^int^ICOS^int^ phenotype that overlapped with the memory chCMV–specific population (Figure 4C).

### CD4^P/I(hi)^ Tfh1 cells infiltrate the liver.

**Figure 5:**
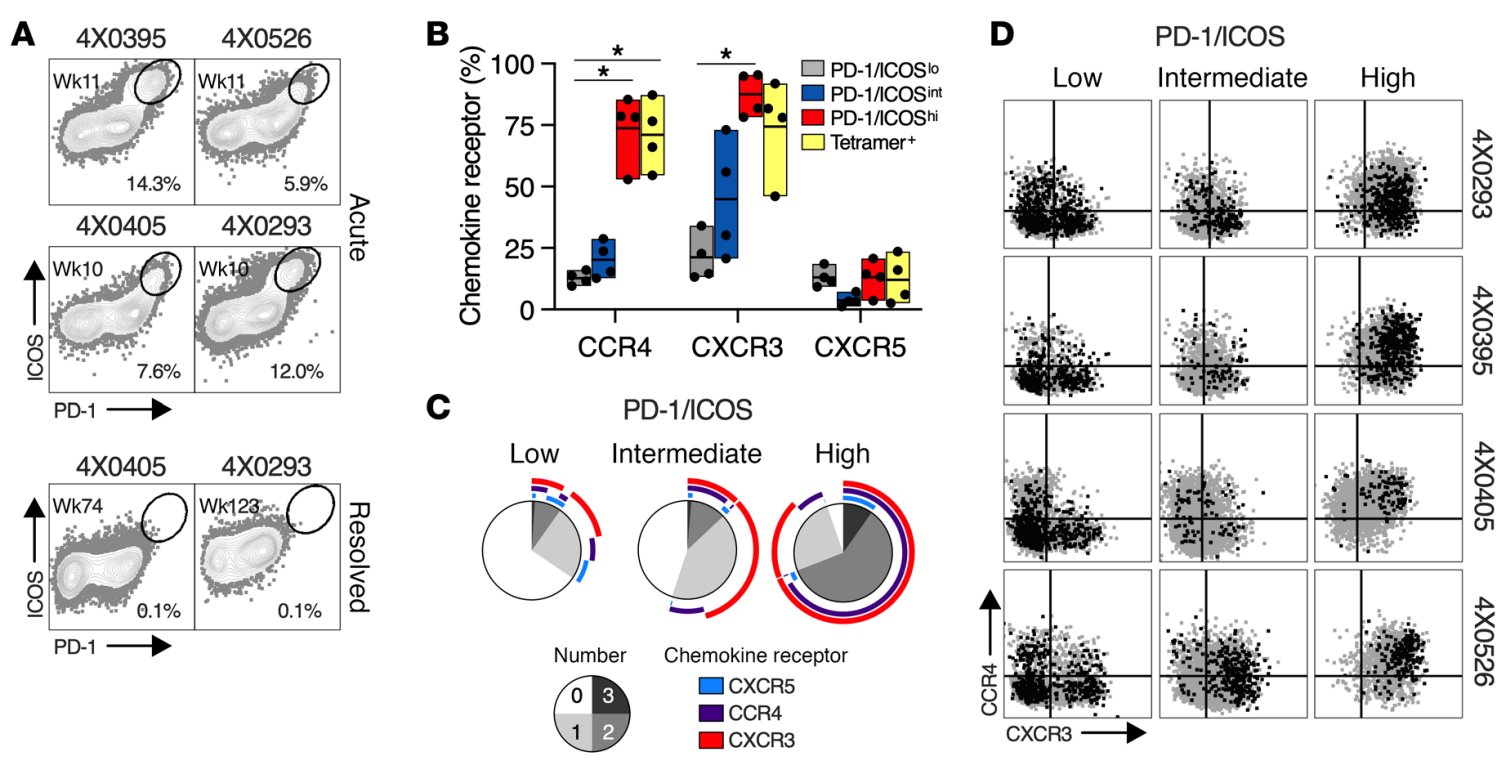
Chemokine receptor expression by liver-infiltrating CD4^P/I(hi)^ T cells. (**A**) Intrahepatic CD4^+^ T cells during acute infection (4X0395, 4X0526, 4X0405, and 4X0293) and approximately 1–2 years after resolution of HCV infection (4X0405 and 4X0293). CD4^P/I(hi)^ T cells were defined by the indicated gate, and percentages are shown. (**B**) Expression of chemokine receptors on intrahepatic CD4^P/I(lo)^, CD4^P/I(int)^, and CD4^P/I(hi)^ T cells, as well as MHC II HCV tetramer–positive CD4^+^ T cells. Each symbol represents 1 animal from **A**. Bars and lines show the range and mean, respectively, of chemokine receptor expression on the indicated CD4^+^ T cell population. Statistical significance (*P < 0.05) was determined by Kruskal-Wallis test with Dunn’s correction for multiple comparisons. (**C**) The number (pie) and combination (arc) of chemokine receptors expressed by CD4^P/I(lo)^, CD4^P/I(int)^, and CD4^P/I(hi)^ T cells in liver, averaged from the 4 animals. (**D**) Intrahepatic CD4^+^ T cell expression of CXCR3 (*x* axis) and CCR4 (*y* axis) with CXCR5 as an overlay (black dots) for the indicated CD4^+^ PD-1/ICOS population.

CD4^P/I(hi)^ T cells were detected in liver at week 10 or 11 after infection (Figure 5A). Frequencies ranged from 6% to 14% of intrahepatic CD4^+^ T cells (Figure 5A), an enrichment of approximately 5- to 10-fold (Supplemental Figure 7A) when compared with the peak in blood that coincided with liver sampling (4X0395) or preceded it by 2–3 weeks (4X0405, 4X0293, and 4X0526) (Supplemental Figure 1, A–D). A similar enrichment in liver was observed for HCV-specific CD4^+^ T cells visualized with class II tetramers (Supplemental Figure 7B). Intrahepatic CD4^P/I(hi)^ T cells represented a response to infection because they were not detected when liver was reassessed in 2 animals approximately 1 (4X0405) to 2 (4X0293) years after termination of viremia (Figure 5A). Almost all intrahepatic CD4^P/I(hi)^ T cells and class II tetramer–positive CD4^+^ T cells expressed chemokine receptors CXCR3 and CCR4 that mediate homing to the HCV-infected liver (Figure 5B) (58–61). CXCR5 was detected on a low frequency of liver-infiltrating CD4^P/I(hi)^ T cells (~12%) and class II tetramer–positive CD4^+^ T cells (~13%) (Figure 5B) in combination with CXCR3 and/or CCR4 (Figure 5, C and D).

**Figure 6:**
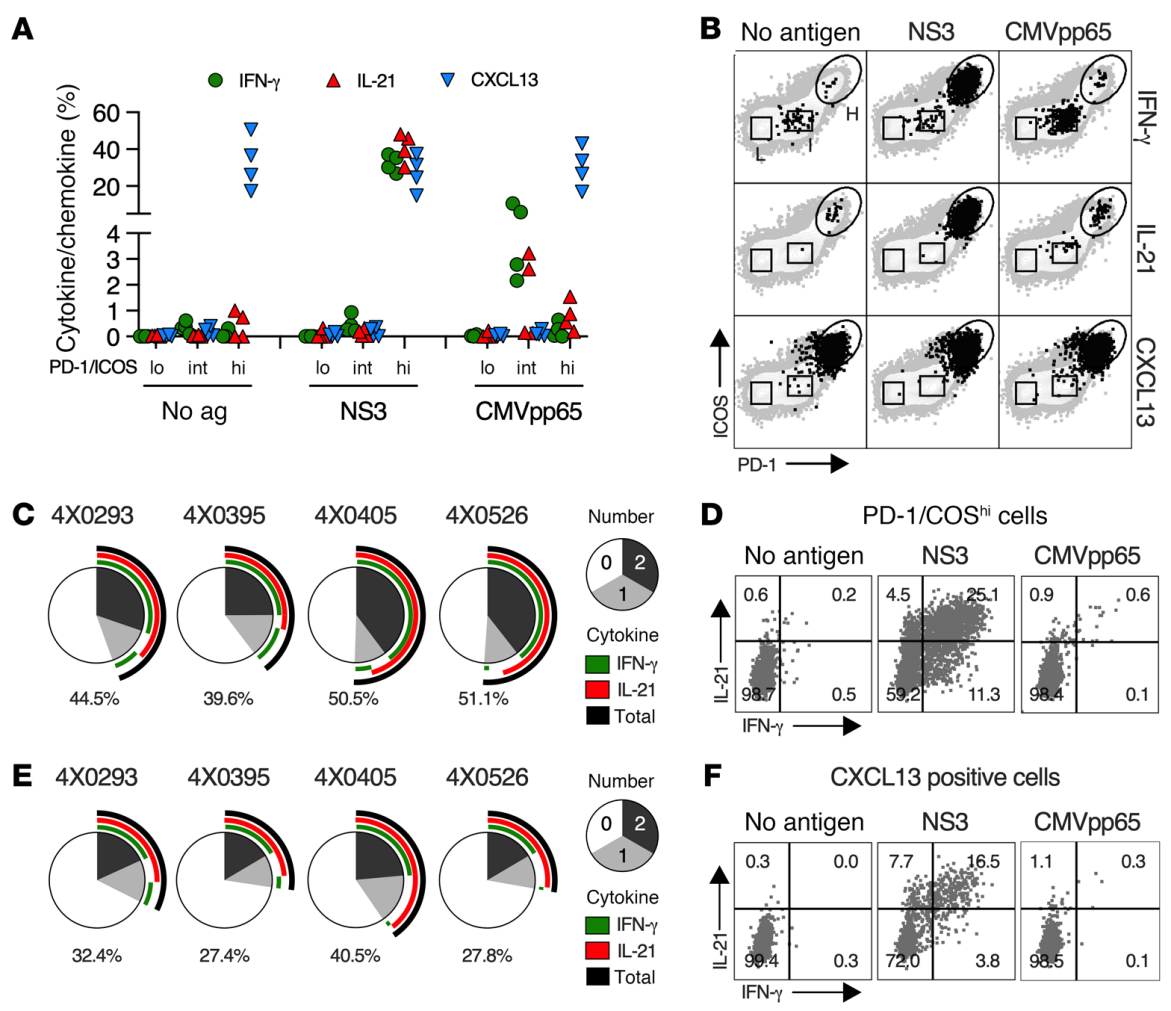
Function of intrahepatic HCV-specific CD4^+^ T cells. (**A**) Percentage of liver CD4^P/I(lo)^, CD4^P/I(int)^, and CD4^P/I(hi)^ T cells positive by ICS for the indicated stimulation condition in 4 animals (4X0293, 4X0395, 4X0405, and 4X0526) during acute infection (Supplemental Figure 1, A–D). Symbols represent individual animals. (**B**) Dot plots with high (H), intermediate (I), and low (L) PD-1/ICOS gates are shown for the representative animal 4X0395. (**C**) The number (pie) and combination (arc) of cytokines produced by CD4^P/I(hi)^ T cells after stimulation with HCV NS3. Black arc and value indicate the percentage of CD4^P/I(hi)^ T cells that produced at least 1 cytokine. (**D**) Cytokine-producing CD4^P/I(hi)^ T cells after stimulation with indicated antigens from 1 representative animal (4X0395). (**E**) The number (pie) and combination (arc) of cytokines coproduced by CXCL13^+^CD4^+^ T cells after HCV NS3 stimulation. The percentage of CXCL13^+^CD4^+^ T cells that produced at least 1 cytokine is shown (black arc and value). (**F**) Dot plots of a representative animal (4X0395) show cytokine-producing CXCL13^+^CD4^+^ T cells after antigen stimulation.

Intrahepatic CD4^+^ T cell function was assessed by ICS (Figure 6, A and B). Approximately 40%–50% of CD4^P/I(hi)^ T cells produced IFN-γ and/or IL-21 after stimulation with NS3, an immunodominant antigen that comprises approximately 20% of the HCV proteome (Figure 6, C and D). Functional chCMV pp65–specific CD4^+^ T cells detected in liver by ICS had the same PD-1^int^ICOS^int^ phenotype as circulating populations (Figure 6, A and B). CXCL13 production by intrahepatic CD4^P/I(hi)^ T cells was again antigen independent, but as in blood they were at least partly HCV specific because approximately 30% (range of 27.4%–40.5%) coproduced IFN-γ and/or IL-21 after stimulation with NS3 but not chCMV pp65 (Figure 6, E and F). In summary, most CD4^P/I(hi)^ T cells that infiltrated liver during acute HCV infection expressed CXCR3 and CCR4, but not CXCR5 which is required for homing to germinal centers. They nonetheless had Tfh1 function defined by coproduction of IFN-γ, CXCL13, and/or IL-21 after HCV NS3 stimulation.

### CD4^P/I(hi)^ Tfh1 cell response to hepatitis A virus infection.

**Figure 7:**
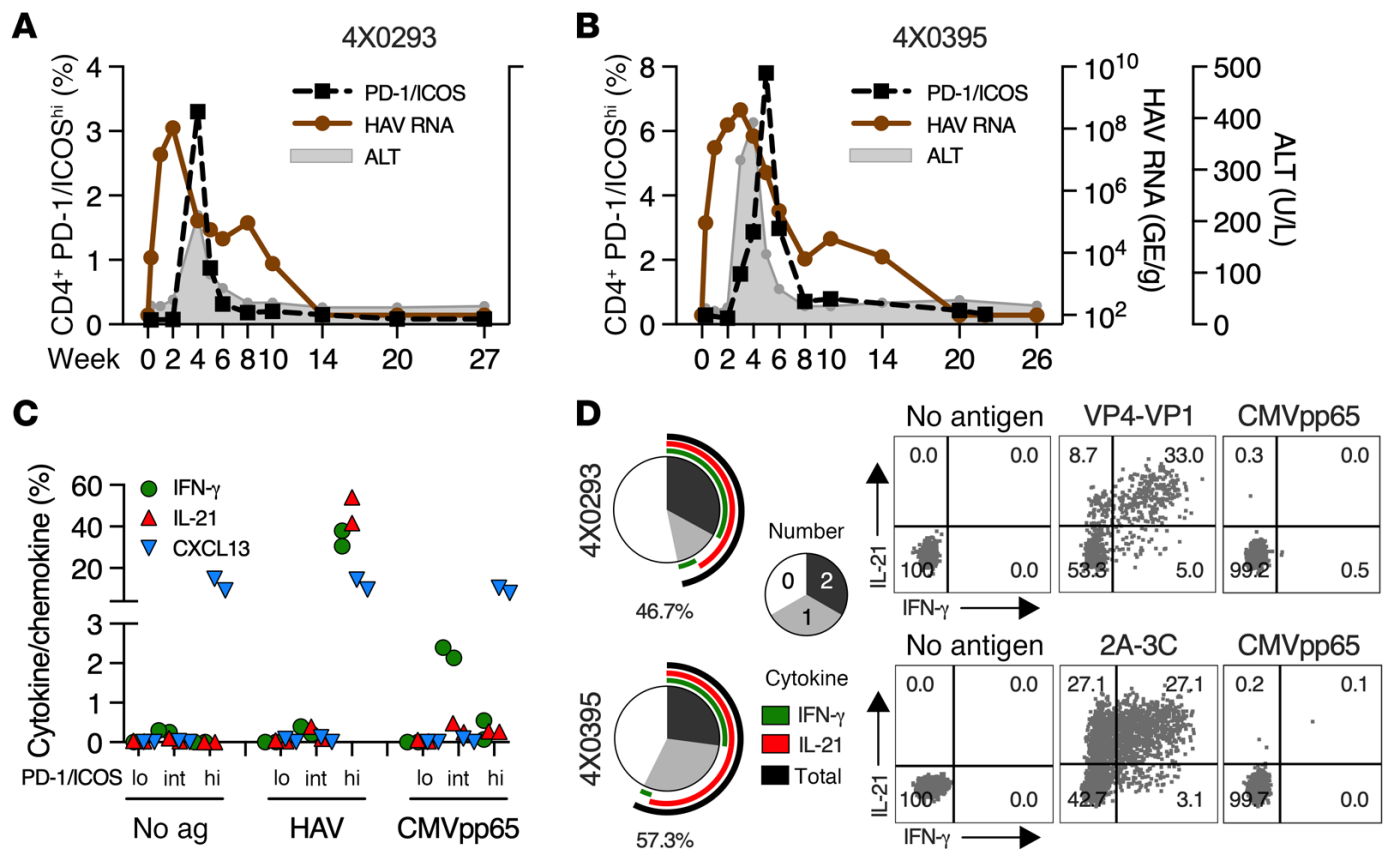
Tfh1 CD4^+^ T cell response in HAV infection. ALT and fecal HAV RNA titers in (**A**) 4X0293 and (**B**) 4X0395 during acute HAV infection as described (62). The frequency of circulating CD4^P/I(hi)^ T cells is also shown. (**C**) Frequency of circulating CD4^+^ T cells at week 4 (4X0293) or week 5 (4X0395) with high, intermediate, or low PD-1 and ICOS coexpression that produced IFN-γ, IL-21, and/or CXCL13 after stimulation with a HAV peptide pool dominant for each animal, as determined previously (62) and shown in Supplemental Figure 8. Cytokine/chemokine production after stimulation with a chCMV pp65 peptide pool is shown for comparison. (**D**) Number (pie) and combination (arc) of cytokines produced by CD4^P/I(hi)^ T cells after HAV antigen stimulation. The percentage of circulating CD4^P/I(hi)^ T cells that produced at least 1 cytokine after antigen stimulation (black arc and value) is shown. Dot plots show IFN-γ and IL-21 production of CD4^P/I(hi)^ T cells following antigen stimulation.

Two animals in this study (4X0293 and 4X0395) resolved acute hepatitis A virus (HAV) infection approximately 2 years before challenge with HCV (62). Control of acute infection was associated with a CD4^+^ T cell response targeting multiple HAV epitopes (62). Here, we demonstrated that this response comprised CD4^P/I(hi)^ T cells that resembled those detected after HCV infection. Circulating CD4^P/I(hi)^ T cells peaked in close temporal proximity to peak ALT (Figure 7, A and B). The HAV-specific helper response was accelerated when compared with acute HCV infection (Figure 2, A and B), as expected given a difference of several weeks in time to onset of adaptive responses and hepatocellular injury in humans and chimpanzees infected with these viruses (1, 16, 63). CD4^+^ T cell function was assessed by ICS after stimulation with dominant HAV peptide pools (VP4-VP1 for 4X0293 and 2A-3C for 4X0395) that each represent approximately 40% of the HAV polyprotein. HAV antigen–dependent production of IL-21 and IFN-γ as well as antigen-independent production of CXCL13 were mediated by CD4^P/I(hi)^ T cells but not CD4^P/I(lo)^ or CD4^P/I(int)^ T cells (Figure 7C). chCMV pp65 stimulation elicited an IFN-γ response by circulating CD4^P/I(int)^ T cells but not CD4^P/I(hi)^ T cells (Figure 7C), as observed during acute HCV infection approximately 2 years later (Figure 4, A and B). Approximately 50% of CD4^P/I(hi)^ T cells produced at least 1 cytokine after stimulation with HAV but not chCMV pp65 antigens (Figure 7D), indicating that they were enriched in HAV-specific populations.

## Discussion

HCV-specific CD4^+^ T cells were assessed in chimpanzees to better understand the complexity of a response ascribed to Th1, Th17, and Tfh subsets that overlap in expression of lineage-defining cytokines, such as IFN-γ and IL-21, and markers, such as CXCR3 (3, 6, 7, 10–12, 30). Class II tetramer–positive CD4^+^ T cells with high PD-1 and ICOS coexpression peaked transiently, but at low frequencies, as expected from other studies in HCV-infected humans and chimpanzees (64). Transcriptional and functional analysis was undertaken instead on a larger population of CD4^P/I(hi)^ T cells that expanded and contracted in parallel with the class II tetramer–positive CD4^+^ T cells. This temporal kinetic overlap suggested that most tetramer-negative CD4^P/I(hi)^ T cells were also HCV specific and not activated bystanders, a possibility supported by several observations. First, approximately 70%–90% of circulating CD4^P/I(hi)^ T cells produced IFN-γ, TNF, and/or IL-21 after direct ex vivo stimulation with NS3-NS5A, HCV antigens that were dominant targets of the CD4^+^ T helper response. CXCL13^+^ CD4^+^ T cells were also predominately HCV specific because most coproduced IL-21, TNF, and/or IFN-γ after stimulation with NS3-NS5A antigens. Second, transcriptional analysis indicated that CD4^P/I(hi)^ T cells were activated, proliferating, and enriched in public TcR clonotypes, as expected for an antiviral response (65, 66). Some upregulated genes, for instance, *BCAT1* and *NFATC2*, are responsive to upstream TcR signaling, suggesting that CD4^P/I(hi)^ T cell expansion was driven by HCV antigen recognition and not non-specific signaling by cytokines or other stimuli. Third, memory chCMV–specific CD4^+^ T cells, a dominant bystander population in blood and liver, did not acquire a PD-1^hi^ICOS^hi^ phenotype or produce CXCL13 during acute HCV infection. We favor the possibility that CD4^P/I(hi)^ T cells that did not respond to NS3-NS5A stimulation (~10%–30% of the total) targeted other structural (core/E1/E2) and nonstructural (NS5B) HCV antigens that were not included in the ICS assay because of limited mononuclear cell availability.

CD4^P/I(hi)^ T cells that expanded in response to HCV infection were Tfh1-like by transcriptional analysis. This hybrid subset assignment was confirmed by immunostaining for Tfh and Th1 transcription factors, cytokines, and chemokine receptors. CXCR5, a chemokine receptor required for Tfh cell migration to germinal centers, was a notable exception. The *CXCR5* gene was differentially expressed by CD4^P/I(hi)^ T cells, but the encoded receptor was detected on only 12% of them by immunostaining, similar to the 13% frequency detected on class II tetramer–positive populations. Most had a CXCR5^–^CXCR3^+^ phenotype that was strikingly similar to CXCR5^–^ Tfh-like cells that infiltrate tumors and tissues inflamed by autoimmune diseases, including the liver (designated T peripheral helper [Tph]) (26, 67–70). Whether Tph-like CD4^+^ T cells that produce CXCL13 are a common feature of virus infections is not known. They were detected in blood during SARS-CoV-2 infection and promoted a plasmablast response, but whether these CD4^+^ T cells were virus specific or infiltrated virus-infected airways was not assessed (71). Here, HCV-specific CD4^P/I(hi)^ T cells that produced CXCL13, IL-21, and IFN-γ were highly enriched in liver, the site of HCV replication. The anatomic location of CD4^P/I(hi)^ T cells remains to be established, but they could contribute to formation of hepatic lymphoid aggregates observed in some patients with acute hepatitis C (72–74). This possibility is supported by CXCL13 production and *SATB1* downregulation, a profile described for CD4^+^ Tph cells that form tertiary lymphoid structures in inflamed tissues and tumors (38, 75). CXCL13-mediated migration of B cells to lymphoid aggregates (76) could explain the significant temporal association between the peak CD4^P/I(hi)^ T cell response and HCV seroconversion observed here. This possibility is supported by detection of IgG^+^ plasma cells in liver lymphoid structures during acute hepatitis C in humans (77). Moreover, an earlier microarray analysis of chimpanzee liver documented upregulation of *CXCL13* and immunoglobulin genes at a time that coincided with the peak CD4^P/I(hi)^ T cell response in this study (78).

CD4^+^ T cells that produce IFN-γ and IL-21 promote CD8^+^ T cell responses and resolution of HCV infection (10). More recently, close proximity of intrahepatic CD8^+^ T cells to Tph-like CD4^+^ T cells that produced IL-21 and CXCL13 was associated with reversal of exhaustion after immune checkpoint inhibitor blockade in hepatocellular carcinoma (79). Here, CD4^P/I(hi)^ T cells also upregulated *LGALS9*, and frequencies in blood were positively associated with serum Gal-9 and CXCL13 titers. Gal-9 costimulates T cells in some models of infection (80, 81), but in acute hepatitis C, it may impair CD8^+^ T cells that express its TIM-3 receptor (10, 33, 82). Kupffer cells produce Gal-9 in the HCV-infected liver (33, 82). This study suggests that CD4^P/I(hi)^ T cells also contribute to its production, possibly in close proximity to intrahepatic CD8^+^ T cells.

CD4^P/I(hi)^ T cells that peaked with ALT upregulated a set of genes associated with cytotoxic function, including several granzymes. However, they did not have the full cytotoxic signature of CD4^P/I(int)^ T cells that upregulated, for instance, *GNLY*, *NKG7*, *PRF1*, and *CX3CR1*. We cannot exclude the possibility that CD4^P/I(hi)^ T cells were directly cytotoxic for hepatocytes, but they may act instead by providing help to CD8^+^ T cells thought to cause hepatocellular injury (83). Other indirect mechanisms of CD4^P/I(hi)^ T cell–mediated immunopathology are also possible. For instance, transcriptional analysis documented upregulation of the proinflammatory cytokine genes *IL1A* and *IL32*, as expected for activated T cells (52, 84). Whether IL-32 contributes to acute hepatocellular injury merits further study given its association with elevated ALT and portal inflammation in chronic HCV infection (32).

PD-1 and ICOS declined on functional HCV-specific CD4^+^ T cells with control of viremia, as observed in humans with resolving infections (29, 30). By week 20 or 24 they had a PD-1^int^ICOS^int^ phenotype equivalent to that measured on memory chCMV–specific CD4^+^ T cells. HCV-specific CD4^+^ Tfh1 cells may undergo further differentiation or repolarization to a more cytotoxic Th17/1 profile as they transition to a PD-1^int^ICOS^int^ phenotype with virus control, although this remains to be established. Tfh1 and Th17/1 CD4^+^ T cells that produce IFN-γ and IL-21 circulate in humans during acute HCV infection (10–12). Whether they represent 1 CD4^+^ T cell population that shifts in Th polarization over time, as suggested by this study, or 2 lineages that develop independently is not known. Further analysis of CD4^P/I(hi)^ T cells in chimpanzees and humans is needed to better define changes in lineage and differentiation as protective memory populations are formed.

This study has limitations. While CD4^+^ T cell responses are very similar in chimpanzees and humans, dominance of CXCR5^–^CXCR3^+^ CD4^P/I(hi)^ T cells that produce CXCL13 during acute HCV infection in humans remains to be confirmed. Additionally, the bulk RNA-Seq approach used here likely underestimated transcriptional complexity because it does not provide insight into coexpression of genes by individual CD4^P/I(hi)^ T cells or differences between the CXCR5^+^ and CXCR5^–^ populations. We also cannot exclude the possibility that some CD4^P/I(hi)^ T cells were activated bystanders, although as noted above a high percentage (~70%–90%) of them were HCV NS3-NS5A specific by ICS. Direct ex vivo HCV antigen stimulation during ICS also had the potential to increase PD-1 and ICOS expression. The stable CD4^P/I(int)^ phenotype of chCMV and late acute-phase HCV-specific CD4^+^ T cells after antigen stimulation argue against this possibility. Finally, our study did not track the onset or fate of CD4^+^ T cell responses in infections that persisted. Although speculative, it is possible that failure of Tfh1 CD4^+^ T cells to shift to a more cytotoxic effector memory Th17/1 profile late in acute infection, when virus replication is often substantially controlled but not eliminated, contributes to persistence. CD4^+^ T cells with a Tfh1 phenotype defined by ICOS, CXCR5, and/or CXCR3 coexpression were observed in the chronically infected liver (11, 85). Transcriptional analysis also demonstrated differential expression of *CXCL13* by circulating HCV-specific CD4^+^ T cells during chronic infection and a decline in this chemokine gene after DAA treatment, possibly reflecting reduced germinal center activity (15). Further study is required to determine if intrahepatic HCV-specific CD4^P/I(hi)^ Tfh1-like cells that produce CXCL13 persist into the chronic phase, where they could contribute to ongoing formation of liver lymphoid structures (85) and high serum CXCL13 titers that decline with DAA treatment (15).

In summary, this study demonstrated an association between expansion of CXCR5^–^ Tfh1-like CD4^+^ T cells, hepatocellular injury, and initial control of HCV replication. These CD4^+^ T cells infiltrated liver and produced CXCL13 that may contribute to formation of lymphoid aggregates observed in the HCV-infected liver. High PD-1 and ICOS coexpression may be a tractable surrogate marker of HCV-specific CD4^+^ T cells that are not easily studied with class II tetramers. Similarly, serum ALT provided a signal for peak expansion in blood and could be used to refine sampling intervals in human subjects. Finally, these findings are likely relevant to development of a protective HCV vaccine and perhaps more broadly to control of infection with other hepatotropic viruses. HCV vaccine development has focused on priming of CD4^+^ Th1 cells given their importance to development of CD8^+^ T cell immunity. This study, and others that described Tfh1-like responses and seroconversion against HCV (11, 12), suggest that vaccine strategies to induce a similar response in blood and possibly liver may be more successful in preventing HCV persistence.

## Methods

### Sex as a biological variable.

Our study examined samples from male (*n* = 6) and female (*n* = 4) animals. While sex-related differences in HCV infection outcome are well established in humans, it was not considered as a biological variable in this study of CD4^+^ T cell immunity. Samples from 10 chimpanzees were included in this study, a large number for this species, but it was not sufficient to assess sex as a biological variable given large individual differences in the time to onset of immunity and infection outcome.

### Samples.

Studies were conducted with cryopreserved serum, blood, and LMC specimens collected as described (17, 62, 86, 87) from 10 HCV genotype 1a–infected adult chimpanzees between May 3, 2005, and May 30, 2013, at the Texas Biomedical Research Institute. Samples were also collected from 2 chimpanzees (4X0293 and 4X0395) during infection with HAV strain HM175 as described (62). The HAV infection resolved 2 years before HCV challenge of these animals.

### Peptide pools.

The 430 peptides (18 aa in length overlapping by 11 aa) were grouped into 9 pools covering HCV WT 1/910 polyprotein sequence for core/E1 (aa 1–389), E2 (aa 379–746), p7/NS2 (aa 736–1,040), NS3-1 (aa 1,030–1,355), NS3-2 (aa 1,345–1,670), NS4A/B (aa 1,660–1,985), NS5A (aa 1,975–2,433), NS5B-1 (aa 2,423–2,734), and NS5B-2 (aa 2,724–3,011). A total of 199 of those peptides were also grouped into 2 pools covering HCV for NS3 (aa 1,030–1,670) and NS4-5A (aa 1,660-2,433). Sixty-eight peptides (18 aa in length overlapping by 10 aa) covering the entire chCMV pp65 sequence were combined into 1 pool. A total of 173 peptides (20 aa in length overlapping by 10 aa) were grouped into 2 pools covering the WT HM175 polyprotein sequence for VP4-VP1 (aa 1–821) and 2A-3C (aa 811–1,751). Peptides were dissolved in sterile water containing 10% DMSO. ICS assays used a final concentration of 2 μg/mL for each peptide.

### Tetramers.

*Patr* class II HCV tetramers (NS3_1248_, NS4b_1761_, NS4b_1825_, and NS4b_1842_) were produced at the NIH Tetramer Facility and are described in Supplemental Table 1. Biotinylated class II monomers were tetramerized at 22°C by adding 20 μL of SA-PE (Agilent) 10 times every 10 minutes for a total of 200 μL SA-PE to 200 g of monomer.

### Antibodies.

Flow cytometry experiments used antibodies for surface markers, chemokine receptors, transcription factors, and intracellular cytokines and chemokines. Antibody details including clone, fluorophore, dilution, and vendor are provided in Supplemental Table 3.

### Flow cytometry assays.

Thawed PBMCs and LMCs were stained with fluorochrome-conjugated antibodies (Supplemental Table 3). All staining and incubations were done at 4°C unless otherwise indicated. Brilliant Stain Buffer (BD Biosciences) was used for antibody dilutions when appropriate. Prior to tetramer staining, cells were blocked with PBS + 20% human serum for 20 minutes. SA-PE–labeled tetramers were diluted 1:200 or 1:400 in FACS buffer (PBS with 2% FBS and 0.1% NaN_3_) and incubated with cells for 30 minutes. Cells were stained with surface antibodies (CD3, CD4, CD8, CD14, CD16, CD19, PD-1, ICOS, CD45RA, and CCR7) for 30 minutes in FACS buffer. Antibody staining for chemokine receptors (CCR4, CXCR3, and CXCR5) was done in PBS with 2% FBS at 37°C for 15 minutes. For ICS, the Intracellular Fixation and Permeabilization Buffer Set (Invitrogen) was used following 16 hours of peptide stimulation of indicated peptide pools at 37°C, 5% CO_2_ in the presence of protein transport inhibitors GolgiPlug and GolgiStop (both BD Biosciences). Permeabilization was done at 22°C for 20 minutes. Cytokines/chemokines (IL-2, IFN-γ, IL-21, TNF, and CXCL13) were stained internally with antibodies diluted in Perm Buffer (Invitrogen) for 30 minutes. Intracellular staining of transcription factors was done using the FOXP3/Transcription Factor Staining Buffer Set (Invitrogen) with a 16-hour permeabilization step and a 3-hour transcription factor antibody stain (BCL-6, BLIMP-1, c-Maf, and T-bet). Viability staining was included in all assays and utilized either Fixable Viability Dye Green or Near-IR (Invitrogen; 1:1,000 dilution in PBS) with 10 or 20 minutes of staining, respectively. Data were acquired on a LSRII or LSR Fortessa cytometer (BD Biosciences) with FACSDiva software (versions 8.0 and 9.0, respectively; BD Biosciences). Cells were sorted using the Influx Cell Sorter (BD Biosciences) with Sortware software (version 1.2.0.142; BD Biosciences). Data were analyzed with FlowJo software (version 10; BD Biosciences).

### RNA-Seq.

Cells were sorted at 4°C by flow cytometry into tubes containing RPMI. Following centrifugation at 600*g* for 10 minutes at 4°C and removal of supernatant, cells were homogenized in 350 μL Buffer RLT (Qiagen) containing 1% 2-mercaptoethanol and extracted using the RNeasy Micro kit (Qiagen) with on-column DNase digestion. RNA quality was assessed using an Agilent 5300 fragment analyzer (Agilent), and 0.5 ng of total RNA was used as input for cDNA synthesis using the Clontech SMART-Seq v4 Ultra Low Input RNA kit (Takara Bio) according to the manufacturer’s instructions. Amplified cDNA was fragmented and appended with dual-indexed barcodes using the Nextera XT DNA Library Preparation Kit (Illumina). Libraries were validated by capillary electrophoresis on a fragment analyzer, pooled at equimolar concentrations, and sequenced on an Illumina NovaSeq 6000 at 100 single reads, yielding 20 million reads per sample. Alignment was performed using STAR version 2.7.3a (88), and transcripts were annotated using the PanTro6 assembly and annotation of the chimpanzee genome (National Center for Biotechnology Information [NCBI] accession GCF_002880755.1). Transcript abundance estimates were calculated internal to the STAR aligner using the htseq-count algorithm (89). DESeq2 was used for normalization (90), producing both a normalized read count table and a regularized log expression table.

TcR sequences were extracted from RNA-Seq reads based on alignment with reference V, D, J, and C genes (GenBank) and were grouped into clonotypes based on CDR3 nucleotide sequence using the software MiXCR (version 4.3.2) through the RNA-Seq pipeline (91). All further analysis was written in R and executed within RStudio (version 4.3.1).

### Chemokine quantification.

Circulating quantities of chemokines were determined using the Bio-Plex Pro Human Chemokine Assay (Bio-Rad) per the manufacturer’s protocol. Briefly, EDTA plasma was thawed on ice, centrifuged at 10,000*g* for 10 minutes at 4°C, and diluted 1:4 with sample diluent HB. Diluted samples, standards, blank, and controls were run in duplicate. Following the antibody staining steps, the assay was read on a Bio-Plex 200 System (Bio-Rad) and analyzed using the Bio-Plex Manager Software (version 6.2; Bio-Rad).

### Gal-9 quantification.

Circulating quantities of Gal-9 were determined using the Human Galectin-9 ELISA Kit (Invitrogen) per the manufacturer’s protocol. Briefly, frozen EDTA plasma was thawed, centrifuged at 10,000*g* for 10 minutes, and diluted 1:5 with 1× assay diluent D. Diluted samples, standards, and blanks were run in duplicate and incubated in the precoated ELISA plate overnight (16 hours) at 4°C with gentle shaking. Following the remaining protocol steps, the assay was read at 450 nm on a BioTek Synergy HTX Multi-Mode Microplate Reader (Agilent) and analyzed using GraphPad Prism software (version 10).

### HCV seroconversion.

Seroconversion to multiple HCV proteins was determined using the Chiron RIBA HCV 3.0 strip immunoblot assay (Novartis Corporation) per the manufacturer’s protocol. Briefly, frozen EDTA plasma was thawed and added to a RIBA strip with specimen diluent. Following a 4-hour incubation at 22°C, the liquid was aspirated, and the strip was incubated for an additional 30 minutes at 22°C in specimen diluent. The strip was washed with working wash buffer and incubated in conjugate for 10 minutes at 22°C. Another wash step was performed, and the strip was incubated for 20 minutes in working substrate at 22°C. After the strip was rinsed with deionized water, it was air-dried in the dark for 1 hour prior to interpretation. The score was determined by comparison of band intensity of HCV antigens and internal controls on each strip.

### Statistics.

Statistical analysis was performed using GraphPad Prism software (version 10) or coded into R using RStudio (versions 4.3.1 and 4.3.2). Statistical significance was calculated by Spearman’s rank correlation, Fisher’s exact test with the Benjamini-Hochberg correction for multiple comparisons, Kruskal-Wallis test with Dunn’s correction for multiple comparisons, Mann-Whitney *U* test, and repeated measures correlation. The statistical test used is indicated in the figure legends. *P* values less than 0.05 were considered statistically significant. All available data were included in the analysis.

### Study approval.

All studies described in this manuscript were funded by the NIH and approved by the Chimpanzee Research Use Panel, Office of the Director, NIH. All samples were collected in accordance with the *Guide for the Care and Use of Laboratory Animals* (National Academies Press, 1996 and 2011) and approved by the IACUC of the Texas Biomedical Research Institute, where the studies were conducted.

### Data availability.

Data values for all graphs are provided in the Supporting Data Values file. RNA-Seq data are available through the NCBI Gene Expression Omnibus (accession GSE247812).

## Author contributions

HB and WB (co–first authors) contributed equally to designing and conducting experiments, acquiring and analyzing data, and preparing figures for publication. HB had primary responsibility for planning and scheduling experiments involving CD4^+^ T cells and is therefore assigned as lead co–first author. YZ, DGB, and RL conducted experiments and established the PBMC and LMC sample bank used in this study. ZX analyzed data (RNA-Seq informatics). CCP and NHS contributed to experimental design and data interpretation. AG managed RNA-Seq experiments and contributed to experimental design. NS analyzed data (TcR sequence analysis). CMW conceived and supervised the study, designed experiments, analyzed data, and wrote the manuscript.

## Funding support

This work is the result of NIH funding, in whole or in part, and is subject to the NIH Public Access Policy. Through acceptance of this federal funding, the NIH has been given a right to make the work publicly available in PubMed Central.

- National Institute of Allergy and Infectious Diseases of the NIH under award numbers R01AI126890, R01AI190069, and U01AI131313 to CMW.
- National Institute of Allergy and Infectious Diseases of the NIH under award numbers R01AI136533 and U19AI159819 to AG and NHS.
- NIH under award number ORIP/OD P51OD011132 (formerly NCRR P51RR000165) to the Emory National Primate Research Center.
- Canadian Institutes of Health Research grant PJT-173467 to NHS.
- NIH P51OD011132 to Yerkes NHP Genomics Core.
- NIH S10 OD026799, for funding of Illumina NovaSeq 6000.

## Supplementary Material

Supplemental data

Supporting data values

## Version 1 09/16/2025

In-Press Preview

## Version 2 11/03/2025

Electronic publication
