# Supplementary material for: A liver-infiltrating CD4+ Tfh1 cell response predicts HCV control, hepatitis, and seroconversion during acute infection
Source: J Clin Invest. 2025 Sep 16;135(21):e178089. doi: 10.1172/JCI178089 (PMC12578392; doi:10.1172/JCI178089)
Supplement: Supplemental data [file jci-135-178089-s044.pdf]

Supplemental Figures 1-8.

Supplemental Tables 1-3.

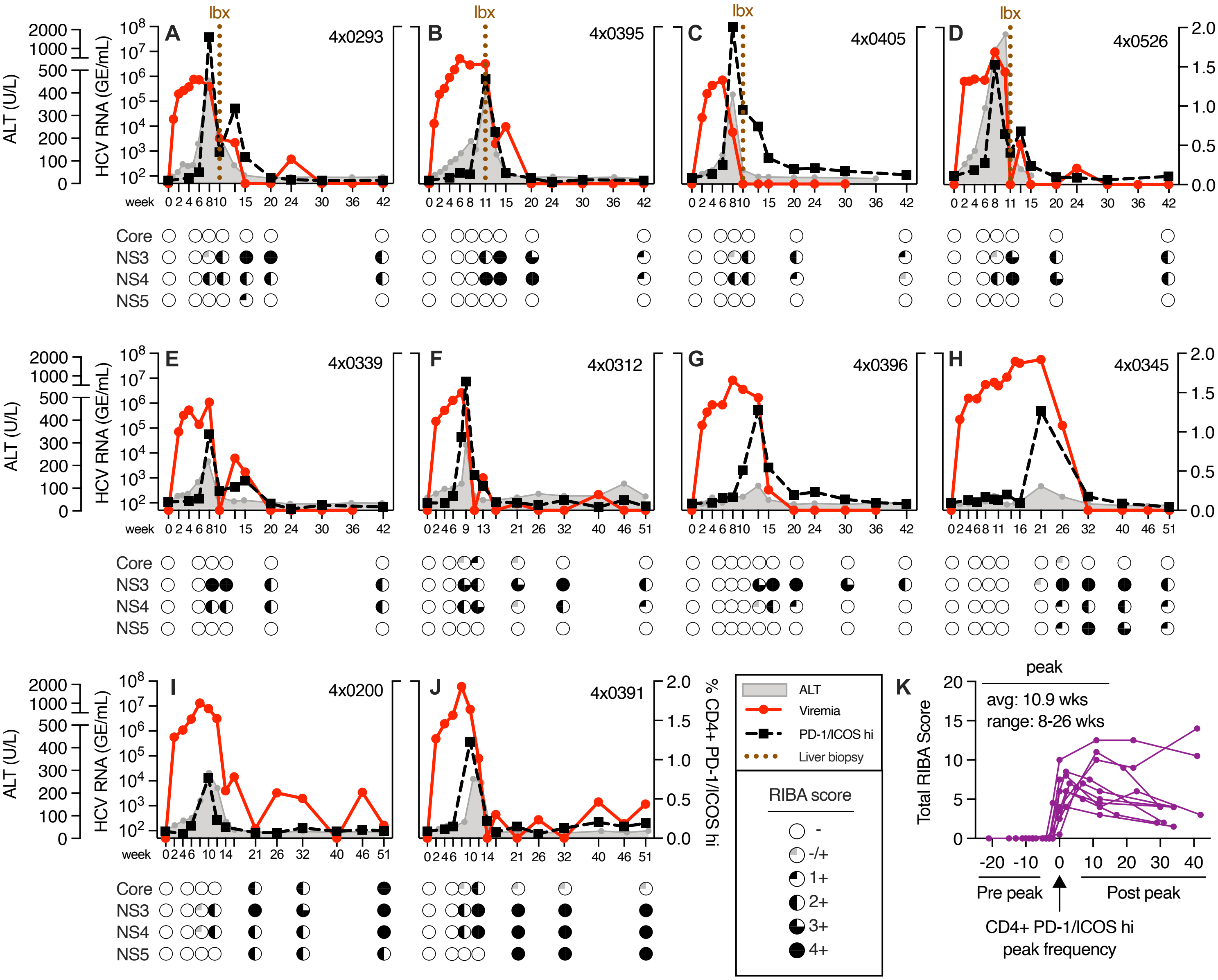

Supplemental Figure 1. (**A-J**) Course of HCV infection in 10 chimpanzees challenged i.v. with a gt1a HCV strain (910). Serum ALT and viremia were measured at the indicated weeks post infection (X-axis). The frequency of CD4^{+} T cells with a PD-1^{hi}ICOS^{hi} phenotype, as defined by gating as shown in Supplemental Figure 2A, was also plotted. The timing of liver sampling is shown for 4 animals used to assess intrahepatic CD4^{+} T cell responses (Panels A-D). Seroconversion to the HCV core and non-structural proteins was assessed at time points aligned with the X-axis of plots A-J using the Chiron recombinant immunoblot assay (RIBA). (**K**) Kinetic of the HCV antibody response in these 10 animals aligned by peak CD4+ PD-1/ICOS high cells. Total RIBA score for each animal is the sum of individual RIBA scores for all 4 assay antigens.

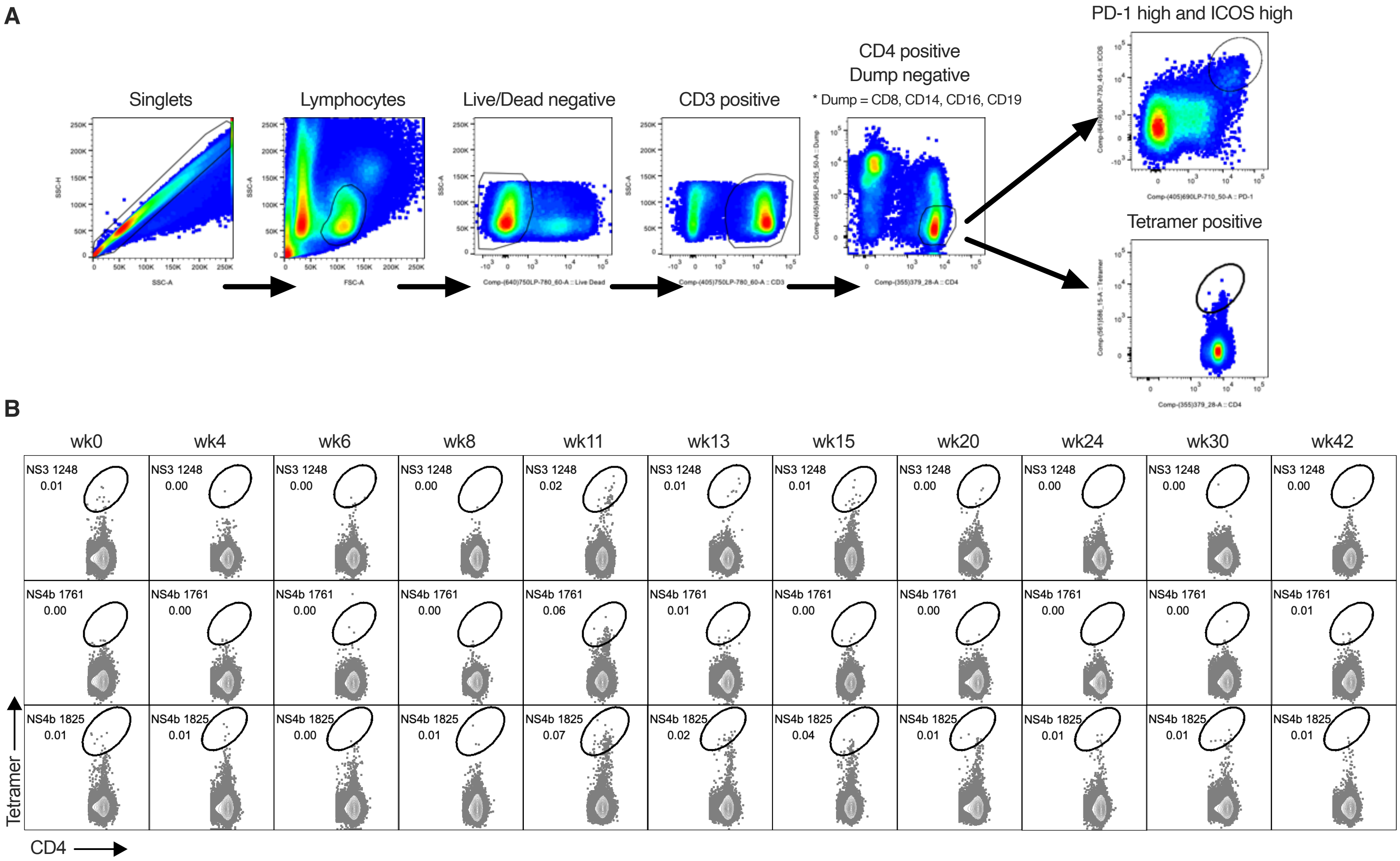

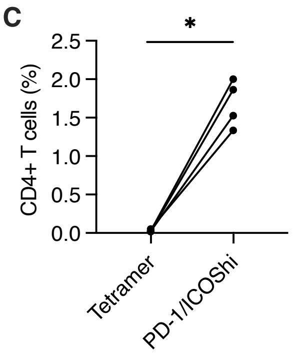

Supplemental Figure 2. (**A**) Gating strategy to identify CD4+ T cells with a PD-1^{hi}ICOS^{hi} phenotype in blood and liver. Plots represent an analysis of circulating CD4+ T cells when PD-1 and ICOS expression peaked in a representative animal (4X0395 wk11). (**B**) Representative staining of PBMC from 4X0395 at the indicated time points with 3 HCV NS3 and NS4B class II tetramers shown in Figure 2 and described in Supplemental Table 1. (**C**) Comparison of circulating CD4+ T cells that were tetramer positive or had high co-expression of PD-1 and ICOS at the peak of response in the 4 animals of Figure 2. Significance was determined by Mann-Whitney u test. *$p < 0.05$.

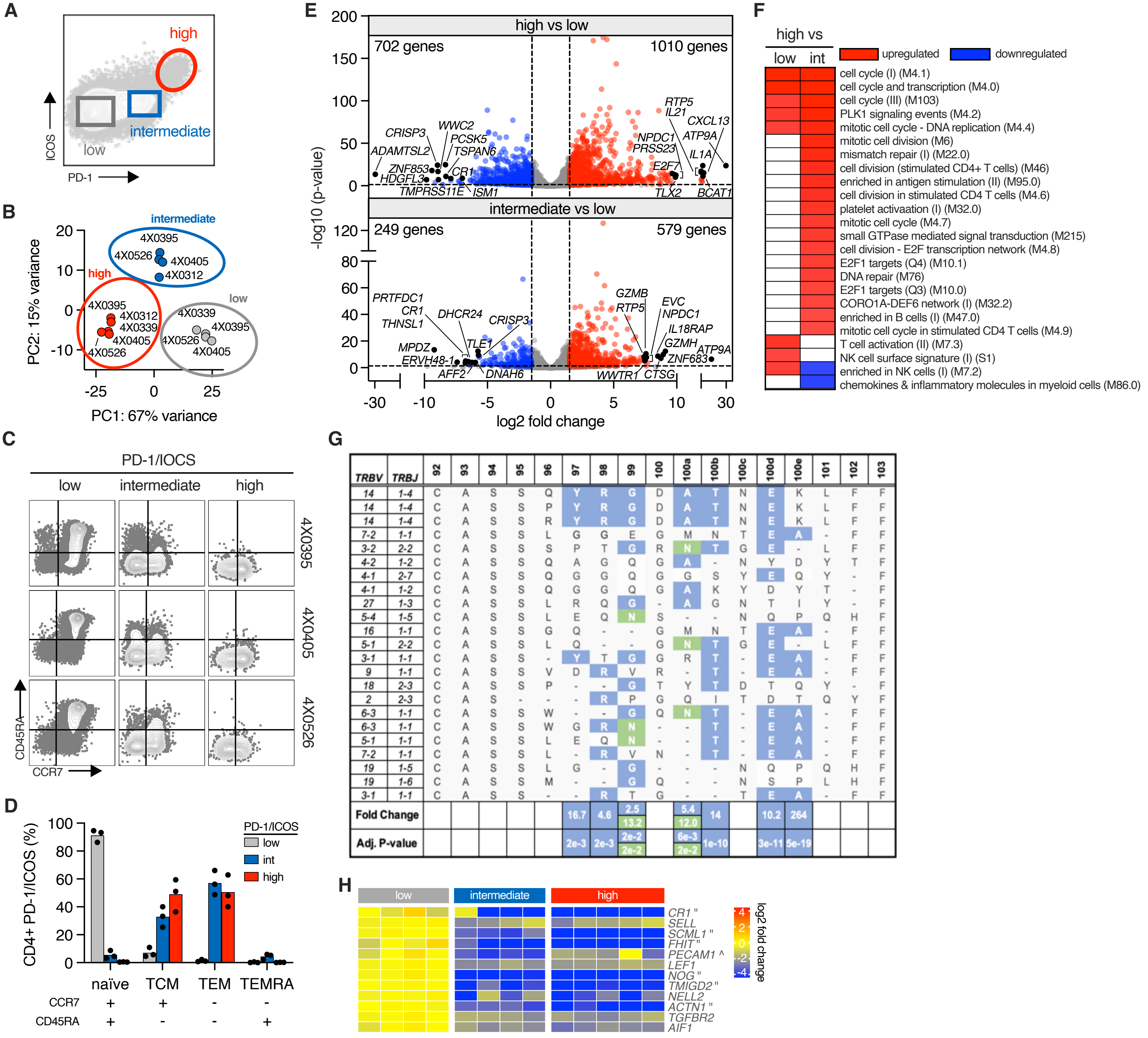

Supplemental Figure 3. (**A**) Gating strategy of CD4+ T cells by low, intermediate, and high PD-1 and ICOS expression, and **(B)** Principal Component Analysis of sorted PD-1/ICOS CD4+ T cell populations. **(C)** Expression of CD45RA versus CCR7 on CD4+ T cells by flow cytometry at the peak of PD-1 and ICOS co-expression (see Figure 2, B-D for details). **(D)** Summary of CD4+ T cells with high, intermediate or low PD-1 and ICOS with a naïve (CCR7+CD45RA+), central memory (TCM; CCR7+CD45RA-), effector memory (TEM; CCR7-CD45RA-), or effector memory re-expressing CD45RA (TEMRA; CCR7-CD45RA+) phenotype. (**E)** Volcano plot of differentially expressed genes (DEG) in a comparison of CD4+ T cells with high versus low PD-1 and ICOS expression, and intermediate versus low expression. The number of genes significantly up- or down-regulated (fold-change >1.5 $\log_2$, padj <0.05) is shown. The top 10 genes in each comparison, ranked by fold change and then significance, are labelled. **(F)** Blood transcriptome matrix (BTM) analysis of CD4+ T cells with a PD-1 and ICOS high phenotype versus CD4+ T cells with low and intermediate phenotypes. **(G)** Alignment of TcRvβ CDRH3 amino acid sequences representing public clonotypes to identify common amino acid motifs. The fold-change and adjusted p-values are shown for significant amino acid positions. **(H)** DEG representative of a naïve status in a comparison of CD4+ T cells with low versus intermediate and low versus high PD-1 and ICOS expression. Top 50 down-regulated genes are indicated for high (*), intermediate (^), or both (") populations.

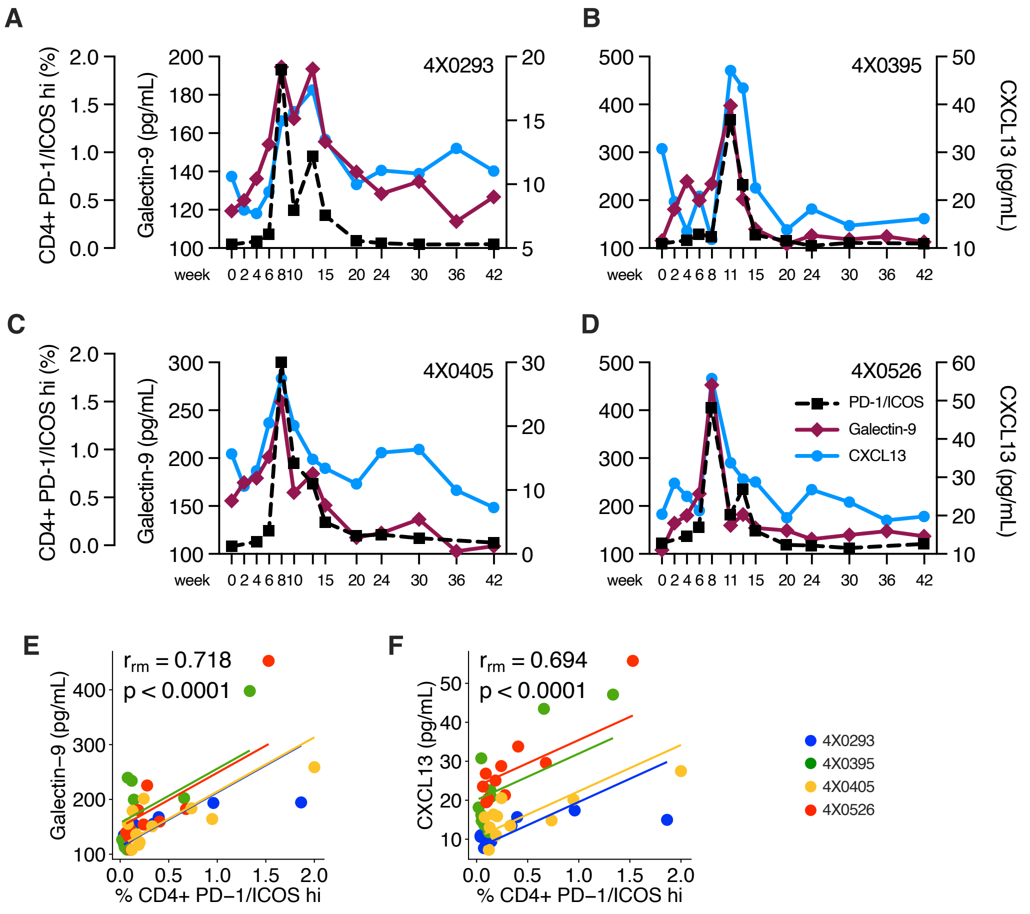

Supplemental Figure 4. (A-D) Serum titers of CXCL13 and galectin-9 in 4 animals at the indicated week post HCV infection. The frequency of circulating CD4+ T cells with a PD-1 and ICOS high phenotype is also plotted. Serum titers of galectin-9 **(E)** and CXCL13 **(F)** versus the frequency of circulating $CD4^{+}$ T cells with a PD-1 and ICOS high phenotype. Significance was established by repeated measures correlation.

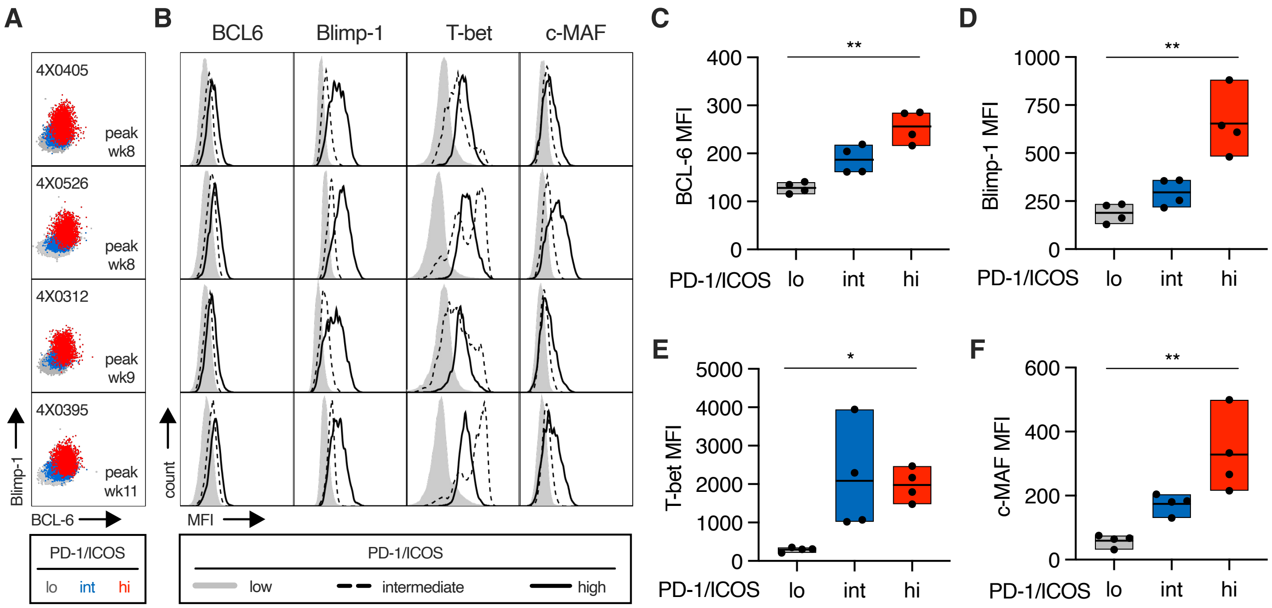

Supplemental Figure 5. (A) Dot plot overlay of BCL-6 and Blimp-1 expression by CD4+ T cells with PD-1 and ICOS low, intermediate and high expression in 4 animals when the PD-1/ICOS response peaked. **(B)** Histogram plot of BCL-6, Blimp-1, T-bet, and c-MAF expression by MFI for CD4+ T cells with PD-1 and ICOS low, intermediate and high expression. Summary of BCL-6 **(C)**, Blimp-1 **(D)**, T-bet **(E)**, and c-MAF **(F)** by CD4+ T cells with the indicated PD-1 and ICOS phenotype. Significance (**p ≤ 0.01, *p ≤ 0.05) was assessed by Kruskal-Wallis test with Dunn's correction for multiple comparisons.

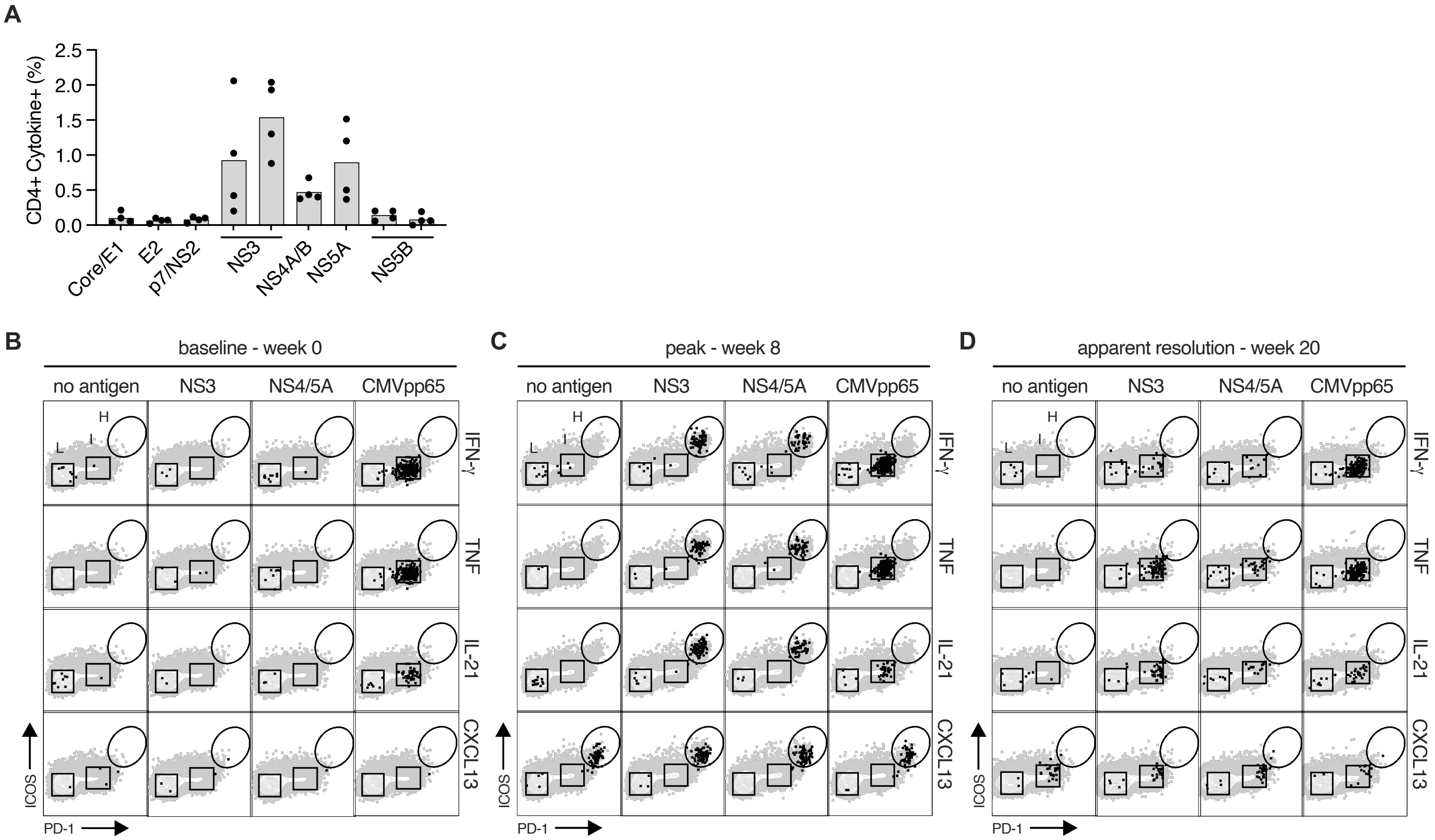

Supplemental Figure 6. (A) Dominance of the CD4+ T cell response against non-structural proteins NS3-NS5A. Frequency of functional intrahepatic CD4+ T cells against the indicated proteins was assessed by ICS in 4 animals at week 10 (4X0405, 4X0293) or 11 (4X0395, 4X0526) p.i. when liver was sampled (Supplemental Figure 1, A-D). Liver mononuclear cells were stimulated with the indicated HCV peptide pool (X-axis) and ICS staining for TNF, IFN-γ, IL-2, and IL-21 was undertaken to determine the frequency of liver CD4$^+$ T cells that produced one or more cytokines against each HCV antigen. PBMC collected from 4X0526 at the week 0 baseline **(B),** week 8 peak of the PD-1^{hi}ICOS^{hi} CD4+ T cell response **(C)**, and week 20 post-infection (**D**) were assessed for cytokine production for the indicated peptide pools. CD4+ T cells positive for IFN-γ, TNF, IL-21, or CXCL13 (black overlay dots) by ICS were localized to the PD-1 and ICOS high, intermediate, and low gates as shown in the dot plots.

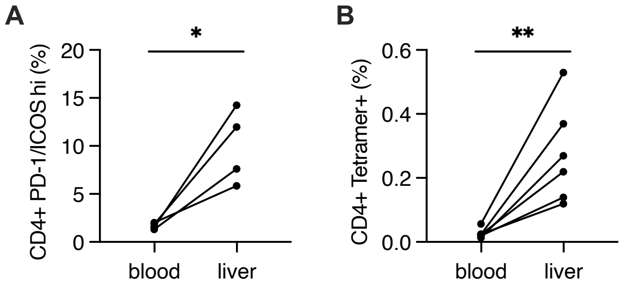

Supplemental Figure 7. Comparison of **(A)** PD-1^{hi}ICOS^{hi} and **(B)** HCV class II tetramer-positive CD4^{+} T cell frequencies in blood at the peak of the PD-1^{hi}ICOS^{hi} response (week 8, 4X0293, 4X0405, 4X0526; week 11, 4X0395) and in liver at the time of sampling (week 10, 4X0293, 4X0405; week 11 4X0395, 4X0526). Frequencies in blood and liver were compared for significance by the Mann-Whitney u test. *$p < 0.05$, **$p < 0.01$.

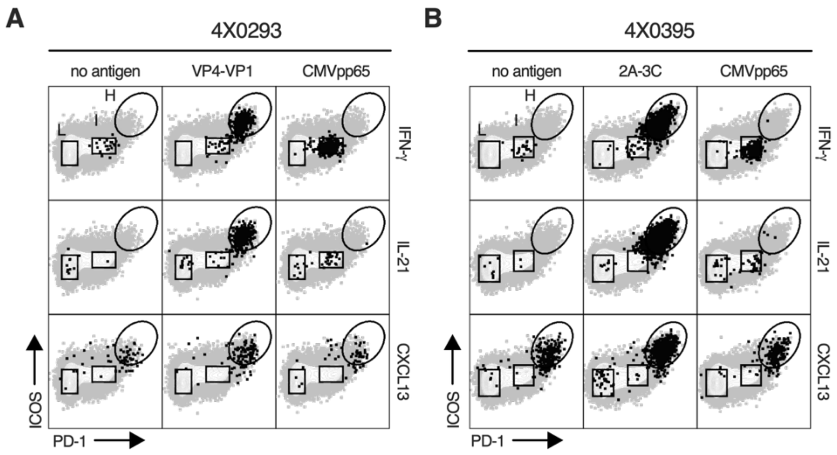

Supplemental Figure 8. CD4+ T cell function was assessed by ICS for **(A)** 4X0293 at week 4 and **(B)** 4X0395 at week 5 p.i. after HAV infection when the circulating PD-1^{hi}ICOS^{hi} CD4+ T cell response peaked in these animals (see Figure 7, A and B). Intracellular staining for IFN-γ, IL-21, and CXCL13 was undertaken with the indicated peptide pools. Cytokine and chemokine producing CD4+ T cells were localized to the PD-1 and ICOS high, intermediate, or low gates as shown on the dot plots.

Supplemental Table 1. ***Patr*** **Class II Tetramers.***

Tetramer	Sequence	Allele
HCV-NS3-1248	GYKVLVLNPSV	Patr-DRB 1*1001
HCV NS4B-1761	WAKHMWNFISGIQYL	Patr-DRB 5*0102
HCV-NS4B-1825	AATAFVGAGLAGAAI	Patr-DRB 5*0102
HCV-NS4B-1842	VGLGKVLIDILAGYGAGV	Patr-DRB 3*0102

*Tetramers are labeled by the location of the epitope (NS3 or NS4B) and the position of the first amino acid in the HCV-1 910 sequence. The amino acid sequence of each epitope (single letter code) and the *Patr* class II allele presenting the epitope are shown.

Supplemental Table S2A. Top 50 upregulated genes for $CD4^{P/I(hi)}$ and $CD4^{P/I(int)}$ T cells versus $CD4^{P/I(lo)}$ T cells.*

	high vs. low		
	Gene	*Log2 FC*	*padj*
	ATP9A	29.9624	2.08E-24
♦	*CXCL13*	12.2019	7.50E-16
♦	*BCAT1*	11.6529	5.30E-12
♦	*IL1A*	11.3441	1.82E-24
	RTP5	10.1237	7.78E-18
♦	*IL21*	10.0742	1.28E-16
	NPDC1	9.9276	4.37E-13
	TLX2	9.9012	4.43E-11
♦	*PRSS23*	9.8336	1.30E-13
♦	*E2F7*	9.6639	4.36E-15
♦	*NR5A2*	9.5298	9.76E-16
	SHOX2	9.3561	2.12E-13
♦	*KIF18B*	9.3179	9.06E-11
♦	*ZNF683*	9.1733	1.25E-12
	TSHR	9.1238	1.92E-10
	ZC3H12C	9.1137	9.58E-14
	CA8	8.9946	2.64E-14
	CKAP2L	8.9849	8.38E-20
♦	*IL10*	8.7933	9.64E-13
	BFSP2	8.6369	1.67E-15
	PDCD1	8.5859	3.32E-89
♦	*MKI67*	8.0828	4.26E-10
♦	*ZBTB32*	8.0806	2.86E-26
	IL7	8.0454	3.33E-10
	CDKN2A	8.0016	2.59E-19
	DTHD1	7.9974	1.67E-34
♦	*E2F8*	7.9718	8.81E-12
	PAQR4	7.8574	5.36E-12
	MYO1E	7.8352	2.56E-10
♦	*ABCB1*	7.1335	2.61E-25
	CAVIN3	7.0135	2.71E-20
	PALM2	7.0029	3.05E-14
♦	*LIF*	6.9984	1.56E-10
♦	*TIGIT*	6.9750	1.84E-27
♦	*GZMK*	6.9672	4.00E-29
♦	*ETV7*	6.9376	4.06E-87
	FAM111B	6.8923	2.12E-13
♦	*IFNG*	6.8781	2.65E-12
♦	*ST8SIA1*	6.8712	1.21E-16
♦	*TK1*	6.8427	4.84E-22
♦	*CD200*	6.8239	3.78E-17
	SYT11	6.7923	1.28E-10
	TBXAS1	6.7414	5.93E-60
	TRIM16	6.7165	1.16E-69
♦	*ASB2*	6.5020	1.15E-76
♦	*CD70*	6.4299	1.19E-19
	KIF11	6.4013	9.80E-49
	MT1E	6.3506	4.68E-33
	CDKN2B	6.3085	6.14E-16
	TMCC2	6.3000	2.47E-51

	intermediate vs. low		
	Gene	*Log2 FC*	*padj*
	ATP9A	18.2571	3.24E-07
♦	*GZMH*	9.1035	3.56E-13
♦	*ZNF683*	8.9306	1.19E-10
	CTSG	8.7488	8.21E-08
♦	*IL18RAP*	8.5016	2.12E-09
	EVC	7.5930	1.26E-08
♦	*GZMB*	7.5626	3.63E-11
	NPDC1	7.4817	4.48E-06
	WWTR1	7.4771	1.06E-06
	RTP5	7.4064	7.03E-09
	CA8	7.1443	2.63E-08
♦	*FASLG*	7.0794	4.72E-06
♦	*GZMK*	6.7381	6.75E-24
	PLEKHA5	6.6583	1.99E-07
	ANKS1B	6.5967	5.18E-06
	EPAS1	6.5922	4.51E-07
	DTHD1	6.5705	1.39E-21
♦	*IL1A*	6.5133	1.09E-07
	FOXF2	6.3670	5.51E-07
♦	*IFNG*	6.3475	1.33E-08
♦	*HHEX*	6.3346	1.44E-15
	ZC3H12C	6.2525	5.38E-06
	C1H1orf21	6.2503	5.79E-06
	CAVIN3	6.2011	2.41E-14
	PDCD1	6.1783	4.00E-38
♦	*ADRB2*	6.1059	1.37E-29
♦	*ABCB1*	5.9586	6.71E-16
	NTRK1	5.9546	1.41E-16
	CGA	5.8897	2.24E-07
♦	*CCL4*	5.4673	1.47E-10
♦	*FGFBP2*	5.4347	9.07E-11
♦	*SPON2*	5.2776	2.38E-12
♦	*GZMA*	5.2276	1.10E-25
	PALM2	5.1756	1.19E-06
♦	*CCL5*	5.1402	1.98E-37
♦	*ADGRG1*	5.1167	5.77E-13
	PATR-DPA1	5.0706	2.41E-18
♦	*NKG7*	5.0384	1.13E-06
♦	*EOMES*	5.0136	3.38E-08
♦	*GNLY*	4.9362	1.30E-09
	SPTB	4.9092	3.53E-07
	APOBEC3G	4.8734	1.10E-45
	AOAH	4.8253	2.98E-11
	GAS7	4.7987	8.45E-31
	GRAMD1C	4.7912	6.73E-07
	PRR5L	4.7609	5.46E-44
	LTK	4.6685	1.52E-07
♦	*ST8SIA1*	4.5797	3.56E-06
	FUT7	4.5785	5.84E-07
	CLIC3	4.4729	2.99E-06

*The top 50 upregulated genes in comparisons of $CD4^{P/I(hi)}$ versus $CD4^{P/I(lo)}$ T cells (left) and $CD4^{P/I(int)}$ versus $CD4^{P/I(lo)}$ T cells (right) were identified by ranking the log_2 fold increase in gene expression followed by p value (padj). Genes discussed in the text and/or presented in Figures 3E and S3H are indicated (♦).

Supplemental Table S2B. Top 50 down-regulated genes for CD4$^{P/I(hi)}$ and CD4$^{P/I(int)}$ T cells versus CD4$^{P/I(lo)}$ T cells.*

high vs. low				intermediate vs low			
	Gene	Log2 FC	padj		Gene	Log2 FC	padj
	ADAMTSL2	-29.6610	3.50E-14		*MPDZ*	-9.2105	3.93E-14
	HDGFL3	-9.8433	7.53E-08		*ERVH48-1*	-7.3996	6.36E-05
	ZNF853	-9.4200	1.38E-18		*THNSL1*	-6.7410	2.15E-04
	CRISP3	-8.9636	4.76E-25	♦	*CR1*	-6.7362	9.63E-06
	WWC2	-8.9381	1.50E-17		*PRTFDC1*	-6.5645	2.23E-05
	TMPRSS11E	-8.9022	7.62E-08		*DHCR24*	-6.4586	5.26E-05
	PCSK5	-8.3368	1.47E-25		*AFF2*	-6.2038	9.34E-05
	TSPAN6	-8.2978	7.40E-12		*DNAH6*	-5.9458	1.04E-04
♦	*CR1*	-7.9056	3.09E-09		*TLE1*	-5.7514	4.71E-13
	ISM1	-7.0058	1.88E-09		*CRISP3*	-5.7011	3.94E-10
♦	*TMIGD2*	-6.1490	3.30E-17		*CARMIL1*	-5.6755	1.27E-08
	PFKFB2	-6.1403	8.38E-10		*MMP28*	-5.6100	4.59E-09
♦	*FHIT*	-5.9183	6.58E-35		*PDE7B*	-5.3516	3.32E-05
	TMEM30B	-5.8968	1.88E-16	♦	*FHIT*	-5.1821	9.41E-24
	TLE2	-5.8691	3.89E-12		*PLAG1*	-5.1129	6.92E-06
	PTK2	-5.5876	4.05E-21	♦	*NOG*	-4.8224	2.70E-21
	VSIG1	-5.5805	8.86E-13		*IGF1R*	-4.7817	7.93E-07
	TTC9	-5.4230	2.40E-20		*ZNF300*	-4.7665	4.29E-04
	OSBPL1A	-5.3945	1.48E-11		*PLLP*	-4.6925	9.29E-07
	CALY	-5.3870	7.32E-09		*PCSK5*	-4.6823	8.44E-12
	NR3C2	-5.3502	8.15E-16		*CACHD1*	-4.4559	2.17E-08
	TLE1	-5.3098	3.65E-14	♦	*TMIGD2*	-4.4414	2.17E-08
	EPHA1	-5.2751	1.87E-20		*UBE2E2*	-4.3790	1.80E-06
	FBP1	-5.2437	4.84E-09		*IQSEC3*	-4.3042	7.83E-09
♦	*NT5E*	-5.2078	3.91E-18		*CLEC4D*	-4.3013	2.36E-04
	RGS11	-5.1971	1.61E-11		*ZNF516*	-3.9445	8.28E-11
♦	*NOG*	-5.1762	4.32E-30		*PRKAR1B*	-3.9100	5.81E-07
	KCNQ1	-5.1149	2.20E-14	♦	*PECAM1*	-3.7470	6.79E-19
	ABCA1	-5.1050	2.37E-20	♦	*HPGD*	-3.6438	2.45E-15
♦	*ZBTB16*	-5.1028	1.57E-16		*MPP1*	-3.5315	8.44E-16
	TBXA2R	-5.0444	8.66E-11		*TSPAN6*	-3.5179	1.55E-04
	GLS2	-5.0441	2.10E-15		*ABCA1*	-3.4716	2.27E-08
♦	*SCML1*	-5.0099	8.52E-90		*ADTRP*	-3.4010	7.39E-08
	TRPC1	-4.9618	2.38E-08		*SLC22A17*	-3.3483	4.70E-11
	MYO15B	-4.9236	1.18E-62		*PTK2*	-3.3238	3.62E-07
	ACTN1	-4.9158	4.84E-64	♦	*SCML1*	-3.2175	3.16E-31
	NLGN2	-4.9105	5.83E-08		*KCTD3*	-3.1560	6.61E-05
	ZNF516	-4.8775	2.28E-19		*ADAMTS6*	-3.0295	7.52E-05
	ARHGEF4	-4.8522	3.10E-08		*CDH4*	-3.0129	2.17E-12
	PDZD4	-4.8450	1.82E-09		*SNPH*	-3.0052	6.76E-10
	PLLP	-4.8423	1.08E-08		*GNAI1*	-2.9705	1.15E-05
	LRP6	-4.7658	1.89E-17		*FAM174B*	-2.9654	2.03E-06
	RNF144A	-4.7284	3.17E-46		*ACTN1*	-2.9027	6.83E-19
	IMMP2L	-4.7253	8.24E-10		*FAM169A*	-2.9016	4.95E-08
	PDGFB	-4.6942	1.94E-15		*PTGIR*	-2.8832	5.00E-10
	CDH4	-4.6547	1.12E-33		*CDCA7L*	-2.8361	1.98E-10
	SYDE2	-4.6514	1.68E-08		*LRP6*	-2.8116	4.58E-06
	RASGRF2	-4.6319	1.94E-49		*C17H17orf51*	-2.7696	5.18E-05
	NOXA1	-4.5409	5.22E-16		*YBX3*	-2.7501	6.34E-17
	LZTFL1	-4.5155	2.12E-14		*CCND1*	-2.6862	7.30E-07

*The top 50 down-regulated genes in comparisons of CD4$^{P/I(hi)}$ versus CD4$^{P/I(lo)}$ T cells (left) and CD4$^{P/I(int)}$ versus CD4$^{P/I(lo)}$ T cells (right) were identified by ranking the $\log_2$ fold decrease in gene expression followed by p value (padj). Genes discussed in the text and/or presented in Figures 3E and S3H are indicated (♦).

Supplemental Table 3. Antibodies used for flow cytometry.

Marker	Clone	Fluorophore	Dilution	Company
BCL6	BCL-UP	APC	1:25	ThermoFisher Scientific
BLIMP1	646702	PE	1:20	R&D Systems
c-MAF	sym0F1	PerCP-eF710	1:25	ThermoFisher Scientific
CCR4	L291H4	BV421	1:25	BioLegend
CCR4	L291H4	BV605	1:25	BioLegend
CCR7	G043H7	BV785	1:25	BioLegend
CD14	M5E2	PE-Cy7	1:50	BD Biosciences
CD14	M5E2	BV510	1:50	BioLegend
CD16	3G8	PE-Cy7	1:200	BD Biosciences
CD16	3G8	BV510	1:50	BioLegend
CD19	HIB19	PE-Cy7	1:50	BD Biosciences
CD19	HIB19	BV510	1:50	BioLegend
CD3	UCHT1	PerCP-Cy5.5	1:20	BD Biosciences
CD3	UCHT1	BV786	1:50	BD Biosciences
CD3	UCHT1	BUV737	1:100	BD Biosciences
CD4	L200	PE	1:5	BD Biosciences
CD4	L200	PE-Cy7	1:200	BD Biosciences
CD4	L200	BV786	1:100	BD Biosciences
CD4	L200	BUV395	1:100	BD Biosciences
CD45RA	HI100	BV510	1:50	BioLegend
CD8	RPA-T8	BV510	1:25	BD Biosciences
CD8	RPA-T8	AF700	1:25	BD Biosciences
CD8	RPA-T8	BUV395	1:100	BD Biosciences
CXCL13	53610	APC	1:25	ThermoFisher
CXCL13	53610	AF700	1:25	R&D Systems
CXCR3	G025H7	PE Dazzle 594	1:50	BioLegend
CXCR5	RF8B2	BB515	1:50	BioLegend
ICOS	C398.4A	FITC	1:50	BioLegend
ICOS	C398.4A	APC	1:100	BioLegend
ICOS	C398.4A	AF700	1:100	BioLegend
IFN-γ	4S.B3	FITC	1:25	BioLegend
IFN-γ	4S.B3	Pacific Blue	1:200	BioLegend
IL-2	MQ1-17H12	APC	1:25	BioLegend
IL-21	eBio3A3-N2	PE	1:30	ThermoFisher Scientific
PD-1	EH12.2H7	BV711	1:50	BioLegend
PD-1	EH12.2H7	BV421	1:20	BioLegend
PD-1	EH12.2H7	BV785	1:20	BioLegend
T-bet	eBio4B10	PE-Cy7	1:200	ThermoFisher Scientific
TNF	Mab11	AF700	1:200	BD Biosciences
